# Plasma Membrane Lipid Asymmetry Dynamically Regulates Passive Diffusion

**DOI:** 10.1002/advs.78435

**Published:** 2026-10-25

**Authors:** Jieming Ping, Wenliang Wang, Jinnuo Xu, Mengyun Yang, Ning Wu

**Affiliations:** ^1^ Department of Immunology School of Basic Medicine Tongji Medical College Huazhong University of Science and Technology Wuhan China; ^2^ The First Affiliated Hospital of Anhui Medical University and Institute of Clinical Immunology Anhui Medical University Hefei China; ^3^ Innovative Institute of Tumor Immunity and Medicine (ITIM) Hefei China; ^4^ Cell Architecture Research Center Tongji Medical College Huazhong University of Science and Technology Wuhan China; ^5^ Anhui Province Key Laboratory of Tumor Immune Microenvironment and Immunotherapy Hefei China; ^6^ Inflammation and Immune Mediated Diseases Laboratory of Anhui Province Anhui Medical University Hefei China

**Keywords:** hydrophobic molecules, passive diffusion, phospholipid asymmetry, ROS, scramblase, scrambling, TMEM16F, XKR8

## Abstract

Plasma membrane (PM) phospholipid asymmetry is a fundamental feature of eukaryotic cells that regulates signaling, trafficking, and cell fate. Whether it also controls passive diffusion across the PM remains unknown. Here, we show that phospholipid asymmetry is a critical determinant of PM permeability to hydrophobic molecules. Using genetic and biochemical approaches, we demonstrate that disruption of lipid asymmetry by the Ca^2^^+^‐activated scramblase TMEM16F or apoptotic scramblase XKR8 profoundly alters the permeation and intracellular accumulation of diverse hydrophobic compounds, including widely used fluorescent probes, while preserving the barrier to membrane‐impermeable molecules. Lipid scrambling is sufficient to remodel membrane permeability to lipophilic molecules, alter membrane biophysical properties, and differentially affect their intracellular accumulation, suggesting selective regulation of passive diffusion rather than nonspecific membrane leakage. Physiological lipid scrambling during T cell activation was also accompanied by altered membrane permeability. Moreover, lipid asymmetry regulates the cellular entry of doxorubicin, 17β‐estradiol, and all‐trans retinoic acid. Together, our findings identify PM lipid asymmetry as a previously unrecognized regulator of membrane permeability and establish a link between membrane organization and molecular transport.

## Graphical abstract

Passive diffusion enables hydrophobic molecules to cross the plasma membrane and exert their biological functions. This study demonstrates that plasma membrane lipid asymmetry, dynamically regulated by lipid scrambling, determines membrane permeability to lipophilic molecules and thereby modulates their cellular activities. Thus, plasma membrane lipid asymmetry is a cell‐intrinsic determinant of membrane permeability.

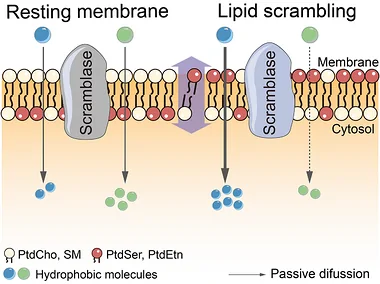

## 1 Introduction

The eukaryotic plasma membrane (PM) is widely viewed as a selective lipid barrier that defines the cellular boundaries and regulates molecular exchange between the intracellular and the extracellular environments [1, 2, 3, 4, 5]. A foundational principle in membrane biology holds that small lipophilic molecules traverse the PM by passive diffusion, with permeability primarily determined by hydrophobicity and solubility, as captured by the classical Overton rule [4, 6, 7]. This view implicitly assumes that the lipid bilayer behaves as a relatively uniform and static phase with respect to diffusion.

However, the PM is not a symmetric or static structure. A defining feature of eukaryotic membranes is the pronounced and actively maintained asymmetry in phospholipids (PLs) distribution between the inner and outer leaflets [5, 8, 9, 10, 11, 12, 13, 14]. The compositional asymmetry arises from the diverse repertoire of lipid species and is dynamically regulated, resulting in a membrane system of remarkable complexity and fluidity [15, 16, 17, 18]. This asymmetry is dynamically established and regulated by lipid flippases, floppases, and scramblases [8, 17, 19]. Beyond its structural role, PM lipid asymmetry is increasingly recognized as a key determinant of cellular physiology and pathology, influencing processes such as signaling, membrane trafficking, and cell fate decisions [10, 18, 19, 20, 21, 22, 23, 24]. However, its fundamental biophysical impact on membrane permeability has remained largely unexplored.

While asymmetry is tightly preserved under homeostatic conditions, its regulated disruption is essential for specific cellular functions [8, 17, 19, 21, 22]. Ca^2+^‐activated scramblase TMEM16F and caspase‐activated scramblase XKR8 are central executors of regulated lipid scrambling. These proteins rapidly collapse membrane PLs asymmetry during diverse biological processes, including platelet activation, maintenance of PM integrity, cell‐cell fusion, viral infection and apoptosis [25, 26, 27, 28, 29]. Despite their well‐established roles in membrane remodeling, it remains unknown whether such dramatic reorganization of lipid distribution influences the basic physicochemical rules governing molecular permeation across the PM.

This question is particularly important because passive diffusion underlies the cellular entry of a wide range of biologically and pharmacologically relevant hydrophobic molecules, including hormones, vitamins, metabolic intermediates, and many clinically used drugs [3, 4, 30, 31, 32, 33, 34]. In addition, the interpretation of cell‐permeable fluorescent probes, ubiquitous tools in modern cell biology, critically depends on stable and predictable membrane permeability [35, 36]. Although passive diffusion represents one of the most fundamental mechanisms of membrane transport discovered long time ago [37, 38], its molecular determinants and regulatory principles in living cells remain poorly defined, particularly in the context of an asymmetrically organized PM [7, 31, 33, 39, 40, 41, 42]. Whether membrane lipid asymmetry modulates the entry and retention of such molecules in living cells has not been addressed.

Here, we propose that PM phospholipid asymmetry is not merely a structural feature but a dynamic biophysical regulator of membrane permeability. We hypothesize that scramblase‐mediated collapse of lipid asymmetry fundamentally alters the physicochemical properties of the lipid bilayer, thereby reshaping passive diffusion of hydrophobic molecules. Using TMEM16F‐ and XKR8‐dependent lipid scrambling as defined and genetically controlled systems, we demonstrate that disruption of PLs asymmetry profoundly and selectively alters cellular permeability to a broad spectrum of hydrophobic molecules, including fluorescent probes, mitochondrial dyes, hormones, and anti‐cancer drugs. Importantly, these permeability changes occur without loss of overall membrane integrity, indicating a regulatory mechanism distinct from membrane rupture or pore formation. Our findings reveal that membrane lipid asymmetry functions as a previously unrecognized, regulable determinant of PM permeability. This work establishes a new conceptual framework in which passive diffusion across the PM is not solely governed by solute hydrophobicity, but is dynamically regulated by the structural state of membrane lipid organization. This has broad implications for membrane biology, drug delivery, and the interpretation of cell‐permeable probes in living cells.

## 2 Results

### 2.1 Collapse of Phospholipid Asymmetry Selectively Reprograms Passive Diffusion Without Disrupting Membrane Integrity

To determine whether plasma membrane lipid asymmetry regulates passive diffusion, we examined the cellular uptake of several widely used cell‐permeable fluorescent probes whose intracellular accumulation depends on membrane permeation. These included the reactive oxygen species (ROS) indicator 2',7'‐dichlorodihydrofluorescein (H2DCFDA, DCF), the protein labeling reagent carboxyfluorescein succinimidyl ester (CFSE), and the nucleic acid dye Hoechst 33342 and Hoechst 33258. As controls, we included the membrane‐impermeable molecules propidium iodide (PI) and dextran‐A488 (Figure 1A). Because these cell‐permeable probes enter cells primarily through passive diffusion and are subsequently retained intracellularly, they provide a sensitive readout of PM permeability [35, 36, 43]. To acutely perturb PM lipid asymmetry, we induced Ca^2+^‐dependent lipid scrambling using ionomycin. Lipid scrambling was verified by Annexin V staining of externalized phosphatidylserine (PS) in living cells that remained impermeable to PI and dextran‐A488. Following ionomycin treatment, RMA cells, a mouse T cell lymphoma line, rapidly underwent robust PS externalization, indicating extensive remodeling of membrane PL organization without detectable loss of membrane integrity (Figure 1B–D). Remarkably, disruption of lipid asymmetry profoundly altered the accumulation of hydrophobic probes. DCF and CFSE fluorescence were markedly lower in scrambled (Annexin V^+^) cells, whereas Hoechst 33342 behaved substantially accelerated nuclear staining, indicating probe‐specific alterations in intracellular labeling of ROS, proteins, and nucleic acids, respectively (Figure 1B–D). Confocal imaging further confirmed that the subcellular localization of both DCF and Hoechst 33342 remained unchanged (Figure S1A). In contrast, dextran‐A488 and PI remained excluded from cells regardless of scrambling status (Figure 1C,D). Thus, collapse of PM asymmetry does not cause nonspecific membrane leakage but instead selectively alters the permeability and accumulation of hydrophobic molecules.

**FIGURE 1:**
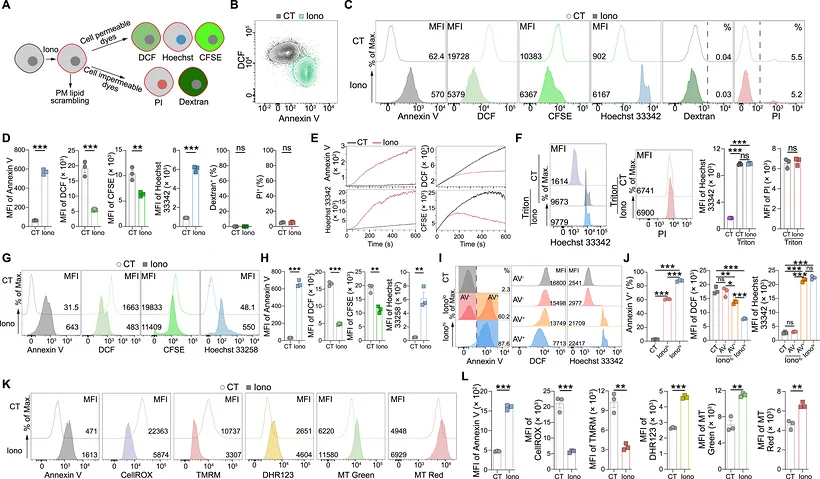
Collapse of phospholipid asymmetry selectively reprograms passive diffusion without disrupting membrane integrity. (A) Schematic illustration of the experimental design. Cells were treated with ionomycin for 5 min and subsequently stained with cell‐permeable or cell‐impermeable dyes for 5 min followed by flow cytometric analysis. (B–D) RMA cells were treated as described in (A). Representative dot plots (B), histograms (C) and quantifications (D) showing PS exposure in living cells and mean fluorescence intensity (MFI) of cell‐permeable dyes, including DCF (10 µM), CFSE (2 µM), Hoechst 33342 (5 µg/mL), or the percentage of cells stained with cell‐impermeable dyes dextran 3kMW (0.1 µg/mL), PI (200 ng/mL) (*n* = 3). (E) Kinetics of Annexin V, DCF, Hoechst 33342, and CFSE staining immediately after ionomycin addition for 10 min. (F) Representative histograms (left) and quantifications (right) of Hoechst 33342 and PI staining in permeabilized RMA cells; CT, untreated control (*n* = 3). RMA cells were treated with ionomycin for 5 min, fixed and permeabilized with 2% PFA and 0.3% Triton X‐100, and stained with PI and Hoechst 33342. (G,H) As in (B–D), representative histograms (G) and quantifications (H) of PS exposure, and the MFI of DCF (10 µm), CFSE (2 µM), Hoechst 33258 (5 µg/mL) in freshly isolated thymocytes (*n* = 3). (I,J) Representative histograms, gating strategy (I) and quantifications (J) showing the percentage of PS exposure and MFI of DCF and Hoechst 33342 (*n* = 3). RMA cells were stimulated with low‐dose (1 µM) and high‐dose (5 µM) ionomycin for 5 min. (K,L) Representative histograms (K) and quantifications (L) of cell‐permeable dyes CellROX (1µM), TMRM (5 µM), DHR123 (5 µm), MitoTracker Green (0.2 µM) and MitoTracker Red (0.2 µM) staining in living cells (*n* = 3). Each dot represents one biological replicate (D,F,H,J,L). Data are presented as mean ± SEM from one representative experiment of two (E,I,K,L), three (F,G), or four (B,C) independent experiments. Statistical analysis was performed using two‐sided unpaired Student's *t* test (D,F,H,L) or one‐way ANOVA (J). *ns*, not significant. ^*^ *p*<0.05, ^**^ *p*<0.01, ^***^ *p*<0.001.

To directly examine the kinetics of this process, Annexin V and fluorescent probes were added simultaneously with ionomycin, and probe accumulation was monitored in real time. PS externalization occurred rapidly following stimulation and was accompanied by an immediate change in probe behavior (Figure 1E). In untreated cells, DCF fluorescence increased steadily over time. By contrast, ionomycin‐treated cells displayed a marked attenuation of DCF accumulation beginning approximately 150 s after stimulation, following the onset of PS exposure (Figure 1E). Similar effects were observed for CFSE (Figure 1E). In contrast, the entry kinetics of Hoechst 33342 and the less permeable analog 33258 were dramatically enhanced following lipid scrambling (Figure 1E and Figure S1B). These findings indicate that changes in membrane phospholipid organization rapidly reshape the diffusion properties of the PM. Importantly, permeabilization of both untreated and ionomycin‐treated RMA cells with Triton X‐100 resulted in comparable labeling of Hoechst 33342 and PI, excluding differences in intracellular DNA content as an explanation for the altered fluorescence signals. (Figure 1F). Instead, these results demonstrate that lipid scrambling primarily affects probe entry across the PM.

To determine whether this phenomenon extends beyond transformed cell lines, we repeated these experiments in freshly isolated mouse thymocytes and splenocytes. Similar to RMA cells, ionomycin‐induced lipid scrambling (Annexin V^+^) in primary cells resulted in markedly less DCF and CFSE accumulation alongside with increased incorporation of Hoechst 33342/33258 (Figure 1G,H, and Figure S1C). Moreover, the magnitude of these effects scaled with ionomycin concentration and the extent of PS exposure (Figure 1I,J), suggesting that PM permeability is quantitatively linked to the degree of asymmetry disruption. Notably, at a lower dose of ionomycin (1 µM), altered DCF and Hoechst 33342 accumulation was restricted to Annexin V^+^ cells, demonstrating that the permeability changes arise from lipid scrambling itself rather than from ionomycin exposure per se (Figure 1I,J). To determine whether the observed changes in fluorescent probe accumulation resulted from active transport or microvesicle shedding, cells were preloaded with CellTrace Violet (CTV, a cytosolic protein‐binding probe) and Calcein‐AM, an intracellular dye commonly used to monitor multidrug resistance (MDR) pump activity, before ionomycin treatment and DCF staining. Confocal imaging and flow cytometry showed that the intracellular signals of CTV and Calcein‐AM were largely retained, whereas DCF accumulation was substantially decreased (Figure S1D,E). To further determine whether microvesicle shedding during ionomycin treatment contributed to the compromised accumulation of hydrophobic probes, we generated GFP‐overexpressing RMA cells and treated them with ionomycin, followed by CTV staining. Notably, less than 10% of the GFP signal was lost, whereas CTV accumulation increased by nearly threefold during this process (Figure S1F). Confocal imaging and GFP immunoblotting further confirmed minimal loss of GFP, accompanied by an increase in cell volume (Figure S1G,H). In addition, we treated cells with ionomycin in the presence of verapamil or zosuquidar, both of which inhibit P‐glycoprotein‐mediated rhodamine 123 (Rho123) efflux. Notably, neither inhibitor significantly affected the accumulation of DCF or Hoechst 33342 (Figure S1I,J), arguing against a major contribution of active efflux to the observed changes in probe accumulation. These results suggest that the accumulation of the hydrophobic molecules is mainly determined by altered membrane permeability and passive diffusion, rather than loss of intracellular contents in our settings.

To further distinguish changes in permeability from potential alterations in intracellular targets, cells were preloaded with DCF, Hoechst 33342/33258 and CTV before induction of lipid scrambling by ionomycin. Under these conditions, DCF fluorescence decreased by approximately threefold in scrambled cells from both RMA cells and primary thymocytes (Figure S1K–M), suggesting reduced intracellular retention or enhanced efflux. In contrast, preloaded CTV or Hoechst 33342/33258 signals remained unchanged following scrambling (Figure S1K,L), confirming that intracellular proteins and nucleic acids were not affected by lipid scrambling. In addition, CTV‐labeled cytosolic proteins, but not Hoechst 33342‐labeled nucleic acids, were lost following membrane permeabilization by Triton X‐100 (Figure S1N). Furthermore, although Hoechst 33342 entered intact cells more efficiently than Hoechst 33258, both dyes labeled nuclei equivalently following complete membrane permeabilization (Figure S1O). Notably, Triton X‐100‐permeabilized cells exhibited higher Hoechst fluorescence than intact cells (Figure S1O), highlighting that PM normally restricts intracellular accumulation of diffusible molecules even in the presence of favorable concentration gradients.

To assess the generality of these findings, we expanded our analysis to structurally diverse cell‐permeable fluorescent probes, including the ROS indicators CellROX and dihydrorhodamine 123 (DHR123), the mitochondrial dyes such as tetramethylrhodamine (TMRM, a cell‐permeant mitochondrial potential‐dependent probe), MitoTracker Green (to label total mitochondria mass) and MitoTracker Red (respiratory mitochondria). Notably, Lipid scrambling consistently altered probe accumulation, although the direction of the effect varied among molecules. CellROX and TMRM behaved similarly to DCF and exhibited reduced staining following scrambling, whereas DHR123, MitoTracker Green, and MitoTracker Red displayed enhanced accumulation under the same conditions (Figure 1K,L). Together, these results reveal that plasma membrane lipid asymmetry is a major determinant of passive diffusion in living cells. Acute collapse of phospholipid asymmetry selectively reprograms the permeability landscape of the PM without compromising membrane integrity, demonstrating that membrane lipid organization functions as a previously unrecognized regulator of hydrophobic molecule transport.

### 2.2 Reduced Accumulation of ROS‐Sensitive Probes Reflects Altered Membrane Permeability Rather Than Diminished Intracellular ROS

The marked reduction in DCF and CellROX staining following lipid scrambling could be interpreted as a decrease in intracellular ROS. However, such an interpretation would be inconsistent with previous studies demonstrating that calcium ionophores elevate intracellular ROS [44, 45]. ROS play essential roles in diverse physiological and pathological processes [46]. However, owing to the chemical heterogeneity and dynamic nature of ROS, their accurate detection in living cells remains technically challenging [47]. Because fluorescent ROS indicators are among the most widely used tools for monitoring cellular redox status, it was critical to determine whether the observed changes in probe fluorescence reflected genuine alterations in ROS levels or instead resulted from altered membrane permeability.

To distinguish between these possibilities, we first employed chloromethyl‐H2DCFDA (CM‐DCF), a derivative of DCF that covalently reacts with intracellular thiol‐containing molecules following cellular entry [48], thereby efficiently retained within the cells (Figure 2A). If lipid scrambling truly reduced ROS production, CM‐DCF fluorescence should decrease following ionomycin treatment. Instead, cells preloaded with CM‐DCF displayed a modest increase in fluorescence after ionomycin stimulation (Figure 2B,C), consistent with previous reports showing that ionomycin elevates intracellular ROS levels [44, 45]. These findings demonstrate that reduced DCF staining in scrambled cells cannot be explained by diminished ROS production. By contrast, when CM‐DCF was applied after the induction of lipid scrambling, probe accumulation was markedly impaired, closely recapitulating the behavior observed for DCF (Figure 2D,E). Thus, the reduced fluorescence signal arises primarily from defective probe incorporation rather than altered intracellular oxidation.

**FIGURE 2:**
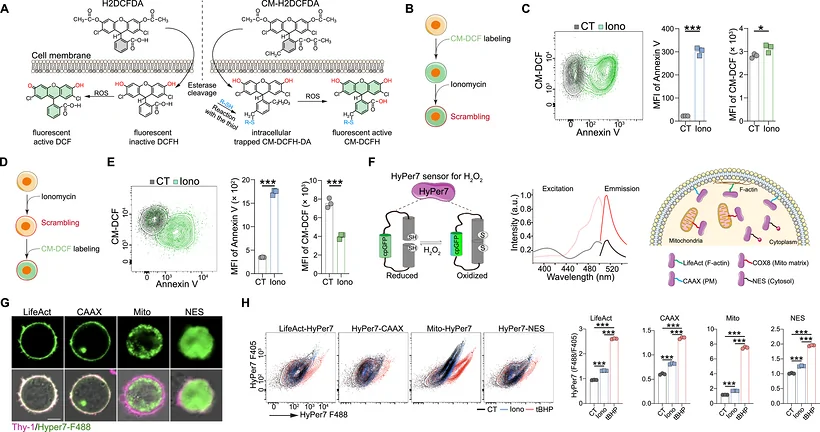
Reduced accumulation of ROS‐sensitive probes reflects altered membrane permeability rather than diminished intracellular ROS. (A) Molecular structures of 2',7'‐dichlorodihydrofluorescein diacetate (H2DCFDA) and 5‐(and‐6)‐chloromethyl‐2',7'‐dichlorodihydrofluorescein diacetate (CM‐H2DCFDA), and their conversion into fluorescent DCF or CM‐DCF for the intracellular ROS detection. (B–E) Schematic illustrations of the experimental design (B,D), representative dot plots (left panels of C,E), and quantification (right panels of C,E) of CM‐DCF staining (*n* = 3). (F) Schematic structure of HyPer7 at reduced and oxidized status (left), excitation and emission spectra of oxidized and reduced HyPer7 (middle), and subcellular localization of LifeAct‐HyPer7 (actin cytoskeleton), HyPer7‐CAAX (inner layer of plasma membrane), Mito‐HyPer7 (mitochondria), and HyPer7‐NES (cytosol) (right). (G) Representative immunofluorescence images showing the localization of HyPer7 variants at the plasma membrane (LifeAct, CAAX), mitochondrial matrix (Mito), and cytosol (NES). Scale bar, 5 µm. (H) Cells in (G) were treated with ionomycin (5 µM, 5 min) or tBHP (10 µM, 10 min). Representative dot plots (left) and quantification (right) of HyPer7 F488/F405 ratio (*n* = 3). Each dot represents one biological replicate (C,E,H). Data are presented as mean ± SEM from one representative experiment of three independent experiments (C,E,H). Statistical analysis was performed using two‐sided unpaired Student's *t* test (C,E), or one‐way ANOVA (H). ^*^ *p*<0.05, ^***^ *p*<0.001.

To directly monitor intracellular ROS independently of membrane permeability, we next utilized HyPer7, a genetically encoded and ratiometric H_2_O_2_ sensor consisting of a circularly permuted GFP (cpGFP) fused to the OxyR regulatory domain from *Neisseria meningitidis* [49]. Upon oxidation by H_2_O_2_, HyPer7 undergoes a shift in excitation from 400 to 499 nm, while maintaining emission at around 516 nm, enabling ratiometric quantification (F488/F405) of intracellular H_2_O_2_ level (Figure 2F). Because HyPer7 is genetically encoded and expressed intracellularly, its signal is unaffected by plasma membrane transport. To examine compartment‐specific ROS dynamics, we generated targeted HyPer7 variants, including LifeAct‐HyPer7 (actin cytoskeleton), HyPer7‐CAAX (inner layer of plasma membrane), Mito‐HyPer7 (mitochondria), and HyPer7‐NES (cytosol) (Figure 2F,G, and Figure S2A). Consistent with previous reports, HyPer7‐positive cells exhibit a robust response to H_2_O_2_ (Figure S2B–D). Strikingly, ionomycin treatment induced a significant increase in HyPer7 oxidation across all examined cellular compartments, although to a lesser extent than the positive control tert‐Butyl hydroperoxide (tBHP) (Figure 2H). These results unequivocally demonstrate that intracellular ROS levels rise rather than decline following ionomycin stimulation. Together, disruption of PM lipid asymmetry limits probe access to the intracellular environment, thereby uncoupling probe fluorescence from the biological process it is intended to measure. More broadly, these results reveal that changes in membrane lipid organization can profoundly influence the interpretation of widely used cell‐permeable fluorescent reporters, highlighting membrane permeability as a previously underappreciated variable in live‐cell imaging experiments.

### 2.3 TMEM16F Establishes a Scramblase‐Dependent Membrane Permeability State

Because ionomycin‐induced lipid scrambling profoundly altered the cellular accumulation of hydrophobic probes, we next sought to identify the molecular mechanism responsible for this permeability switch. Ca^2+^‐activated PM lipid scrambling is mediated by TMEM16F, an evolutionarily conserved Ca^2+^‐dependent scramblase [19, 26, 50, 51, 52]. We therefore hypothesized that TMEM16F‐dependent remodeling of lipid asymmetry underlies the observed changes in membrane permeability. To test this hypothesis, we generated TMEM16F‐deficient cell lines using CRISPR‐Cas9 technology, including RMA (mouse lymphoma), YAC‐1 (mouse thymoma), EL4 (mouse lymphoma), L1210 (mouse leukemia), and Jurkat (human T cell lymphoma) cells (Figure 3A and Figure S3A–D). Consistent with our previous observations, ionomycin robustly induced PS exposure in wild‐type (WT) RMA cells but failed to trigger lipid scrambling in TMEM16F knockout (KO) cells (Figure 3B–E). Strikingly, loss of TMEM16F completely abolished the permeability changes associated with lipid scrambling (Figure 3B–E and Figure S3E,F). Notably, whereas WT cells displayed reduced intracellular accumulation of DCF, CellROX, and CFSE following ionomycin treatment, TMEM16F‐deficient cells retained probe incorporation at levels comparable to untreated controls (Figure 3B–E and Figure S3E,F). Conversely, TMEM16F‐mediated scrambling dramatically enhanced accumulation of Hoechst 33342 and the less permeable analog Hoechst 33258 in WT cells, whereas this effect was entirely absent in TMEM16F‐deficient cells (Figure 3D,E and Figure S3G,H). Real‐time kinetic analyses of Annexin V binding, DCF fluorescence, and Hoechst uptake further corroborated these findings, further linking membrane permeability changes to TMEM16F‐dependent scrambling (Figure S3I). Several observations excluded alternative explanations based on differences in intracellular probe targets. Pre‐staining experiments demonstrated comparable Hoechst 33258 fluorescence in both WT and TMEM16F KO RMA cells (Figure S3J), indicating equal amounts of nucleic acids in both cells, and excluding the possibility of DNA loss following ionomycin‐induced lipid scrambling. Moreover, complete permeabilization with Triton X‐100 resulted in similar maximal Hoechst staining in WT and KO cells despite marked differences in probe uptake kinetics before permeabilization (Figure S3K). Thus, TMEM16F does not alter intracellular binding capacity but instead regulates access of hydrophobic molecules across the PM.

**FIGURE 3:**
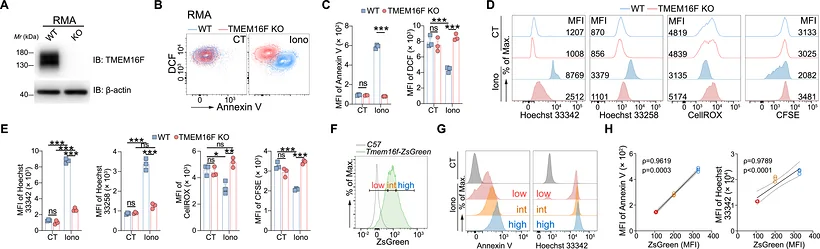
TMEM16F establishes a scramblase‐dependent membrane permeability state. (A) Immunoblot of TMEM16F expression in RMA cells. β‐actin served as the loading control. Original blot images are provided in (Figure S11A). (B,C) WT and TMEM16F KO RMA cells were treated with ionomycin (5 µM) and stained with cell‐permeable dyes DCF (10 µM) for 5 min. Representative dot plots (B) and quantification (C) of PS exposure and DCF fluorescence in living cells (*n* = 3). (D,E) As in (B,C), representative histograms (D) and quantification (E) of the MFI of Hoechst 33342, Hoechst 33258, CellROX and CFSE in WT and TMEM16F KO cells following ionomycin treatment (*n* = 3). (F–H) Gating strategy for ZsGreen fluorescence in thymocytes (F), categorized as low, intermediate, or high populations using a 25% cut‐off. Representative histograms (G) showing the Annexin V and Hoechst 33342 staining in living cells after ionomycin treatment, and Spearman correlation analysis (H) between ZsGreen and Annexin V or Hoechst 33342 (*n* = 3). Each dot represents one biological replicate (C,E,H). Data are presented as mean ± SEM from one representative experiment of at least two independent experiments (B to H). Statistical analysis was performed using two‐way ANOVA (C,E), or Spearman correlation analysis (H). *ns*, not significant. ^*^ *p*<0.05, ^**^ *p*<0.01, ^***^ *p*<0.001.

Importantly, the relationship between TMEM16F activity and membrane permeability was not restricted to a single cell type. Similar TMEM16F‐dependent effects on DCF, CFSE, CellROX, and Hoechst incorporation were observed in primary thymocytes isolated from WT (*Tmem16f^+/+^*) and TMEM16F KO (*Tmem16f^−/−^*) mice (Figure S3L–N). Likewise, attenuation of DCF accumulation following lipid scrambling was observed in multiple murine cell lines, including EL4, YAC‐1 and L1210 cells (Figure S4A–C). In the human T cell line Jurkat, TMEM16F deficiency similarly abolished scrambling‐induced enhancement of Hoechst uptake (Figure S4D,E). Although the behavior of DCF and CFSE exhibited some cell type‐specific variation (Figure S4D,E), TMEM16F‐dependent lipid scrambling consistently altered the permeability of hydrophobic molecules across all mouse and human systems examined.

To further investigate whether membrane permeability scales with the extent of TMEM16F activity, we took advantage of *Tmem16f‐ZsGreen* reporter mice [53], in which endogenous TMEM16F expression can be monitored by ZsGreen fluorescence. Thymocytes were stratified into low, intermediate, and high TMEM16F‐expressing populations based on ZsGreen fluorescence intensity (Figure 3F). Notably, the degree of ionomycin‐induced PS externalization increased with TMEM16F expression and was accompanied by a corresponding increase in Hoechst 33342 incorporation (Figure 3G,H). Thus, membrane permeability is not merely influenced by the presence or absence of TMEM16F but quantitatively tracks with the magnitude of TMEM16F‐mediated lipid scrambling.

Together, these findings identify TMEM16F as a central molecular regulator of PM permeability. By controlling the extent of phospholipid asymmetry, TMEM16F establishes distinct membrane permeability states that regulate the passive diffusion and accumulation of hydrophobic molecules. These results provide direct genetic evidence that lipid asymmetry is mechanistically coupled to membrane transport properties in living cells.

### 2.4 TMEM16F‐Mediated Lipid Scrambling Is Sufficient to Reprogram Membrane Permeability

Having established that TMEM16F is required for scrambling‐dependent changes in membrane permeability, we next asked whether lipid scrambling itself is sufficient to generate these permeability states. To address this question, we performed a series of genetic rescue and gain‐of‐function experiments designed to uncouple lipid scrambling from ionomycin treatment and directly test the contribution of TMEM16F activity. Re‐expression of full‐length (FL) mouse TMEM16F in TMEM16F KO RMA cells using a retroviral system, largely restored PS externalization following ionomycin stimulation (Figure S5A–C,I). Importantly, restoration of lipid scrambling was accompanied by reduced DCF accumulation and enhanced uptake of Hoechst 33342, thereby recapitulating the phenotype observed in WT cells (Figure S5B,C). In contrast, the scrambling‐defective mutant, arginine mutated to alanine at position 478 (R478A), failed to induce PS exposure and did not restore the reduction of DCF staining characteristic of scrambling‐competent cells (Figure S5B,C,I). Although R478A partially enhanced Hoechst uptake, its inability to fully reproduce the permeability phenotype indicates that efficient lipid scrambling is required for complete remodeling of membrane transport properties.

Conversely, ectopic expression of the gain‐of‐function mutant F518A restored PS exposure and resulted in reduced DCF and CellROX staining alongside enhanced Hoechst 33342 incorporation (Figure S5D–H). Thus, permeability changes consistently tracked with the ability of TMEM16F variants to scramble membrane phospholipids.

To further test whether scrambling activity itself is sufficient to confer the permeability phenotype, independent of the specific TMEM16F protein backbone, we exploited chimeric proteins between TMEM16F and the closely related family member TMEM16A, which lacks phospholipid scrambling activity. Substitution of the TMEM16F scrambling domain (SCRD) into TMEM16A (AFA chimera) [52], a family member lacking lipid scrambling activity, conferred the ability to externalize PS in TMEM16F‐deficient cells (Figure S6A–C). Acquisition of scrambling activity was accompanied by reduced DCF uptake and enhanced Hoechst 33342 entry (Figure S6B,C). In contrast, replacement of the TMEM16F SCRD with the corresponding region from TMEM16A (FAF chimera) abolished lipid scrambling activity and failed to alter either DCF or Hoechst staining (Figure S6B,C). These results demonstrate that TMEM16F‐mediated lipid scrambling is both necessary and sufficient to modulate the cell membrane permeability to hydrophobic molecular probes.

To determine whether lipid scrambling is sufficient to alter membrane permeability in the absence of ionomycin, we next examined constitutively active TMEM16F mutants. Replacement of phenylalanine by histidine at position 518 (F518H) in TMEM16F has been reported as a gain‐of‐function mutation that confers spontaneous lipid scrambling activity [54]. Consistent with this notion, expression of F518H in TMEM16F‐deficient cells induced basal PS exposure in the absence of stimulation (Figure S6D–I). Following Triton X‐100 permeabilization, F518H‐expressing cells exhibited DNA content comparable to TMEM16F KO cells, indicating no loss of intracellular nucleic acids (Figure S6J). Despite F518H‐expressing cells displayed only partial spontaneous scrambling (around 15%, compared to ∼ 2% in TMEM16F KO cells), they displayed markedly altered permeability properties, including enhanced accumulation of DCF and Hoechst dyes (Figure S6D,E). Similar results were obtained with the constitutively active D430G‐L mutant [55], which likewise induced spontaneous PS externalization and altered uptake of hydrophobic probes without ionomycin treatment (Figure S6F–I). These findings indicate that remodeling of membrane permeability does not require acute Ca^2+^ influx or ionophore treatment. Rather, alteration of phospholipid asymmetry alone is sufficient to establish distinct permeability states.

Consistent with these alterations, lipid scrambling in WT cells was associated with pronounced changes in membrane biophysical properties, including membrane rigidity and fluidity, assessed by Laurdan dye and merocyanine 540 (MC540) staining [56, 57, 58]. In contrast, TMEM16F KO cells exhibited only modest changes in membrane properties upon ionomycin treatment (Figure S6K–N). These observations suggest that TMEM16F‐mediated redistribution of phospholipids fundamentally reorganizes membrane architecture and thereby modifies the energetic landscape governing passive diffusion.

Collectively, these experiments establish that TMEM16F‐mediated lipid scrambling is both necessary and sufficient to remodel PM permeability. By reorganizing phospholipid asymmetry and membrane biophysical properties, TMEM16F generates distinct permeability states that selectively regulate the diffusion of hydrophobic molecules independent of ionomycin stimulation or global membrane disruption.

### 2.5 Disruption of PM Asymmetry Broadly Remodels Permeability to Hydrophobic Molecules

Our preceding results demonstrated that TMEM16F‐mediated lipid scrambling affects the cellular uptake of several commonly used fluorescent probes. We next asked whether this phenomenon reflects a probe‐specific effect or instead represents a broader change in plasma membrane permeability. To address this question, we expanded our analysis to additional classes of hydrophobic molecules with distinct intracellular targets and physicochemical properties. We first examined mitochondrial fluorescent probes, including MitoTracker Green and MitoTracker Red, as well as TMRM, a mitochondrial potential‐dependent dye. Strikingly, MitoTracker Green and Red exhibited elevated staining in WT cells undergoing scrambling upon ionomycin treatment, whereas no significant changes were observed in TMEM16F‐deficient cells (Figure 4A–C). Importantly, preloading experiments revealed comparable MitoTracker fluorescence between WT and TMEM16F KO cells regardless of ionomycin treatment, excluding differences in mitochondrial abundance as an explanation for the altered staining patterns (Figure 4D–F). Thus, changes in probe accumulation primarily reflect altered membrane permeability rather than changes in organelle content.

**FIGURE 4:**
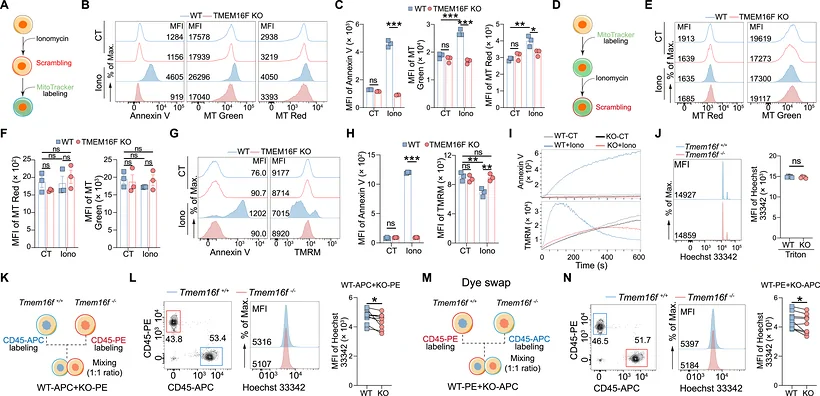
Disruption of PM asymmetry broadly remodels permeability to hydrophobic molecules. (A–F) Schematic illustrations of experimental design (A,D), representative histograms (B,E) and quantifications (C,F) of PS exposure and intracellular fluorescence levels of MitoTracker Green (MT Green) and MitoTracker Red (MT Red) in living cells (*n* = 3). (G,H) Representative histograms (G) and quantification (H) of PS exposure and MFI of TMRM in WT and TMEM16F KO cells following ionomycin treatment (*n* = 3). (I) Kinetics of Annexin V and TMRM fluorescence immediately after ionomycin addition for 10 min. (J) Representative histograms (left) and quantification (right) of intracellular Hoechst 33342 fluorescence in permeabilized WT and TMEM16F KO thymocytes (*n* = 5). (K–N) Schematic illustrations of experimental design (K,M). WT and TMEM16F KO thymocytes were labeled with anti‐CD45‐APC or anti‐CD45‐PE antibody, respectively, mixed at a 1:1 ratio, and stained with Hoechst 33342 (5 µg/mL) for 20 min. Dye labels were swapped in the second experiment (N). Gating strategy (left panels of L,N), representative histograms (middle panels of L,N) and quantification (right panels of L,N) of Hoechst 33342 fluorescence (*n* = 9). Each dot represents a biological replicate (C,F,H) or one individual mouse (J,L,N). Data are presented as mean ± SEM pooled from two independent experiments (L,N), or from one representative experiment of at least two independent experiments (B,C,E–J). Statistical analysis was performed using two‐sided paired Student's *t* test (L,N) or unpaired Student's *t* test (J), or two‐way ANOVA (C,F,H). *ns*, not significant. ^*^ *p*<0.05, ^**^ *p*<0.01, ^***^ *p*<0.001.

In contrast, TMRM exhibited reduced accumulation in PS‐externalizing WT cells, but remaining largely unchanged in TMEM16F KO cells (Figure 4G,H). Kinetic analysis revealed a rapid initial increase in TMRM signal following ionomycin treatment, reaching a peak at approximately 100 s, followed by a progressive decline that ultimately fell below the levels in untreated controls after 10 min (Figure 4I). To further distinguish the ionomycin‐induced mitochondrial potential damage from the reduction in TMRM fluorescence, we performed a prolonged TMRM staining. As expected, both WT and TMEM16F KO cells exhibited a comparable decrease in TMRM fluorescence (Figure S7A). Although mitochondrial membrane potential likely contributes to these dynamics, the TMEM16F dependence of the response further supports the conclusion that lipid scrambling profoundly influences the intracellular accumulation of structurally diverse hydrophobic molecules.

To assess whether lipid scrambling affects the selective permeability without compromising the fundamental barrier function of the PM, we examined the behavior of Alexa Fluor 488‐labeled dextran, a membrane‐impermeable molecule. Consistent with our previous observations, dextran‐A488 remained excluded from both WT and TMEM16F KO cells regardless of ionomycin treatment (Figure S7B). Conversely, dextran‐A488 introduced into cells by electroporation was efficiently retained following lipid scrambling, with no evidence of dye leakage (Figure S7C), further supporting the conclusion that membrane barrier function remains intact. These findings demonstrate that collapse of phospholipid asymmetry does not generate nonspecific membrane rupture or leakiness. Instead, lipid scrambling selectively remodels permeability to hydrophobic molecules while preserving overall membrane integrity.

Because TMEM16F is generally regarded as a stimulus‐dependent phospholipid scramblase, we next asked whether it contributes to membrane permeability under basal conditions. To minimize experimental variation, we mixed thymocytes from WT (*Tmem16f* ^+/+^) and TMEM16F KO (*Tmem16f* ^−/−^) mice in the same tube and distinguished by differential anti‐CD45 antibodies labeling (e.g., APC and PE). Remarkably, TMEM16F‐deficient thymocytes consistently exhibited reduced incorporation of Hoechst 33342 compared with WT cells despite equivalent DNA content (Figure 4J–N). Similar results were obtained in RMA cells as well (Figure S7D,E). These results indicate that TMEM16F influences membrane permeability even in the absence of experimental stimulation.

Together, these findings demonstrate that TMEM16F‐mediated lipid scrambling disrupts the PM asymmetry and broadly modulates the permeability of hydrophobic molecules. Moreover, the altered permeability observed in TMEM16F‐deficient cells under resting conditions suggests that TMEM16F contributes to the establishment of basal membrane transport properties. These results identify PM lipid asymmetry as a previously unrecognized modulator of passive diffusion and reveal TMEM16F as a physiological regulator of membrane permeability beyond its established role in stimulus‐induced phospholipid scrambling.

### 2.6 Apoptotic Lipid Scrambling Mediated by XKR8 Remodels Membrane Permeability Through a Convergent Mechanism

Collapse of membrane phospholipid asymmetry is a hallmark of apoptosis. Our findings with TMEM16F suggested that disruption of PM phospholipid asymmetry can profoundly alter the permeability of hydrophobic molecules. To determine whether the apoptosis‐associated lipid scrambling similarly altered the membrane permeability, we employed raptinal, a potent apoptosis inducer via mitochondrial pathways [59]. Importantly, RMA WT cells exhibited robust PS exposure and displayed reduced staining of DCF and enhanced accumulation of CTV. However, treatment with MDR pump inhibitors or inhibitors of membrane blebbing did not alter the fluorescence signals following raptinal‐induced PS exposure, arguing that the observed changes in fluorescence primarily reflect altered membrane permeability resulting from lipid scrambling (Figure S8A,B). In addition to Ca^2+^‐activated lipid scrambling mediated by TMEM16F, apoptotic lipid scrambling is governed by XKR8 [29]. We further ablated *Xkr8* expression in RMA cells using CRISPR‐Cas9 technology (Figure S8C–E). Upon induction of apoptosis by raptinal, WT RMA cells exhibited robust PS exposure, whereas XKR8‐deficient RMA cells failed to do so (Figure 5A,B). Notably, WT cells undergoing XKR8‐dependent scrambling displayed reduced staining of DCF and enhanced accumulation of Hoechst 33342/33258 following apoptosis induction, whereas these effects were largely absent in XKR8 KO cells (Figure 5A,B). Thus, two mechanistically distinct scrambling pathways converged on a remarkably similar permeability phenotype. Importantly, intracellular DNA content remained comparable between WT and XKR8 KO cells and was not changed by raptinal treatment, as shown by equivalent Hoechst 33342 staining following permeabilization by Triton X‐100 (Figure 5C). Kinetic analyses further revealed that Hoechst 33342/33258 uptake closely paralleled Annexin V binding in WT cells, but not in XKR8‐deficient cells (Figure 5D and Figure S8F). Moreover, within WT populations, Annexin V^+^ subset exhibited markedly faster incorporation of Hoechst 33258 than Annexin V^−^ cells (Figure S8G,H), reinforcing the association between PS exposure and enhanced probe entry across the PM. In addition to Hoechst dyes, apoptotic lipid scrambling in WT RMA cells significantly disturbed the behavior of other hydrophobic probes. CFSE and TMRM staining were substantially reduced following raptinal treatment in WT cells (Figure 5E,F). Although partial reductions were also observed in XKR8‐deficient cells (Figure 5E,F), likely reflecting scrambling‐independent consequences of apoptosis, the more pronounced effects observed in wild‐type cells indicate a major contribution from XKR8‐mediated loss of membrane asymmetry. To determine whether the observed changes in TMRM fluorescence reflected alterations in mitochondrial membrane potential or plasma membrane permeability, we preloaded WT and XKR8 KO cells with TMRM before raptinal treatment. Notably, both WT and XKR8 KO cells exhibited similarly low TMRM fluorescence (Figure S8I). These results indicate that raptinal treatment compromised mitochondrial membrane potential independently of XKR8 and support the conclusion that XKR8‐mediated lipid scrambling directly alters plasma membrane permeability to hydrophobic compounds.

**FIGURE 5:**
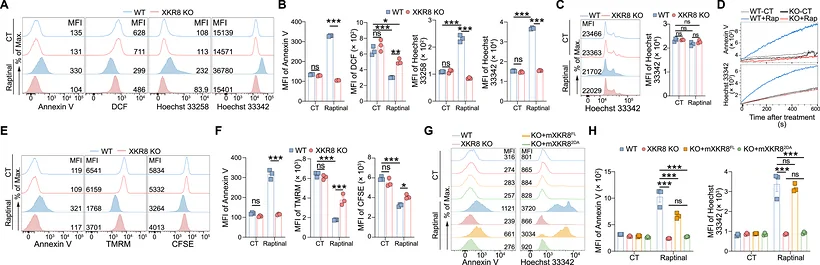
Apoptotic lipid scrambling mediated by XKR8 remodels membrane permeability through a convergent mechanism. (A,B) WT and XKR8 KO RMA cells were treated with raptinal (10 µm) for 1 h and subsequently stained with cell‐permeable dyes for 5 min. Representative histograms (A) and quantifications (B) of PS exposure, DCF, Hoechst 33258/33342 fluorescence in living cells (*n* = 3). (C) Representative histograms (left) and quantifications (right) of intracellular Hoechst 33342 in permeabilized WT and XKR8 KO cells following raptinal treatment (*n* = 3). (D) Kinetics of Annexin V and Hoechst 33342 fluorescence recorded for 10 min after 1 h raptinal treatment in WT and XKR8 KO cells. (E,F) Representative histograms (E) and quantifications (F) of PS exposure and MFI of TMRM and CFSE in living cells following raptinal treatment (*n* = 3). (G,H) As in (A,B) representative histograms (G) and quantifications (H) of PS exposure and Hoechst 33342 in WT, XKR8 KO and XKR8 KO cells reconstituted with full‐length XKR8 or the 2DA mutant (*n* = 3). Each dot represents one biological replicate (B,C,F,H). Data are presented as mean ± SEM from one representative experiment of at least two independent experiments (A to H). Statistical analysis was performed using two‐way ANOVA (B,C,F,H). *ns*, not significant. ^*^ *p*<0.05, ^**^ *p*<0.01, ^***^ *p*<0.001.

Activation of XKR8 requires caspase‐3‐mediated cleavage at the DLVD motif (position 351–354 amino acids) [29, 60]. Re‐expression of full‐length mouse XKR8 in XKR8 KO RMA cells successfully restored PS externalization upon raptinal treatment and resulted in a marked increase in Hoechst 33342 incorporation (Figure 5G,H and Figure S8J,L). However, ectopic expression of a cleavage‐deficient mutant (2DA, replacement of aspartic acid by alanine at position 351 and 354 amino acid) failed to rescue either lipid scrambling or permeability changes (Figure 5G,H and Figure S8J,L), demonstrating that activation of the XKR8 scrambling machinery is required for membrane remodeling. Furthermore, introduction of constitutively active XKR8 mutants (N371A and F381A) [61] in the TMEM16F and XKR8 double KO RMA cells (DKO) induced spontaneous lipid scrambling in the absence of apoptosis (Figure S8N,O), while reintroduction of these mutants reinstated PS exposure during raptinal‐induced apoptosis and confocal images confirmed their PM localization (Figure S8K–M). Importantly, Annexin V^+^ subsets displayed significantly increased uptake of Hoechst 33258 and compromised staining of DCF, demonstrating that loss of PM asymmetry by XKR8‐mediated lipid scrambling is sufficient to remodel membrane permeability independently of the broader apoptotic program (Figure S8N,O). Collectively, these findings establish that XKR8‐mediated apoptotic lipid scrambling, analogous to TMEM16F‐dependent scrambling, regulates plasma membrane permeability to hydrophobic molecules, highlighting loss of phospholipid asymmetry itself as the critical determinant of the permeability state and establishing lipid scrambling as a general mechanism linking membrane organization to passive diffusion.

### 2.7 Lipid Scrambling During T Cell Activation Is Associated With Altered PM Permeability

Loss of PM asymmetry through lipid scrambling is not restricted to programmed cell death but also occurs in physiological contexts such as immune cell activation [8, 21, 22, 62]. Upon activation, naïve T cells undergo a profound metabolic reprogramming characterized by increased oxidative phosphorylation (OXPHOS), generating large amount of ATP and accompanied by increased ROS [63, 64, 65, 66, 67]. During this highly anabolic state, T cells require substantial uptake of extracellular nutrients [66, 68, 69]. While most metabolites rely on dedicated transporters, certain lipophilic molecules, including fatty acids and lipid‐soluble vitamins essential for T cell function, can enter cells through passive diffusion [70].

Although lipid scrambling during CD8^+^ T cell activation has been reported previously and implicated in regulating CD8^+^ T cell exhaustion during chronic LCMV infection [71, 72, 73], its functional consequences on PM transport properties remain unclear. Based on our previous findings, we hypothesized that activation‐associated loss of PM asymmetry may modulate PM permeability to hydrophobic molecules. To test this, CD8^+^ T cells were activated by stimulating freshly isolated splenocytes with anti‐CD3ε monoclonal antibody for 6 h, with successful activation confirmed by upregulation of the activation markers CD69 and CD25 (Figure 6A,B). A robust scrambling state was detected in activated CD8^+^ T cells, as indicated by Annexin V binding (Figure 6C,D). In parallel, elevated DCF fluorescence was observed, consistent with increased intracellular ROS production during activation (Figure 6C,D). Notably, Hoechst 33342 uptake was significantly accelerated in activated CD8^+^ T cells compared with unstimulated controls, with Annexin V^+^ cells exhibiting higher Hoechst signals than Annexin V^−^ cells (Figure 6C–E). Importantly, intracellular DNA content remained unchanged after 6 h of stimulation, as assessed by Hoechst 33342 staining of permeabilized CD8^+^ T cells (Figure 6F).

**FIGURE 6:**
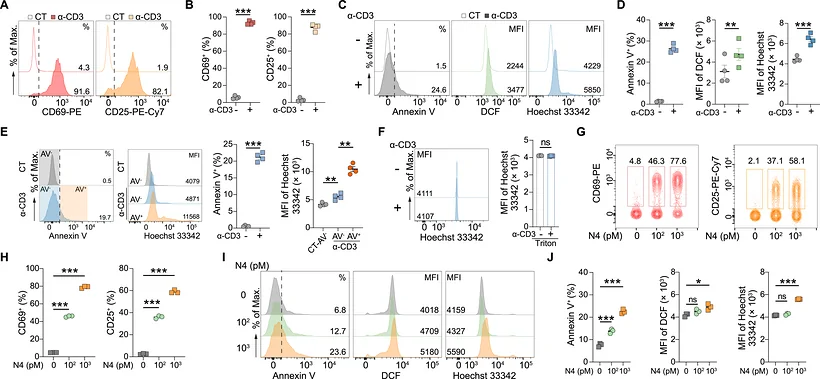
Lipid scrambling during T cell activation is associated with altered PM permeability. (A,B) Splenic CD8^+^ T cells were stimulated by anti‐CD3ε (0.1 µg/mL) for 6 h. Representative histograms (A) and quantifications (B) of CD25 and CD69 levels on CD8^+^ T cells (*n* = 4). CT, unstimulated control. (C–E) Activated CD8^+^ T cells were stained with Annexin V and cell‐permeable dyes DCF (10 µM) and Hoechst 33342 (5 µg/mL) for 10 min. Representative histograms (C) and quantifications (D) of PS exposure and MFI of DCF, Hoechst 33342 in activated splenic CD8^+^ T cells. Hoechst 33342 fluorescence in Annexin V^+^ and Annexin V^−^ populations of living cells (E) (*n* = 4). (F) Representative histogram (left) and quantification (right) of Hoechst 33342 levels in permeabilized splenic CD8^+^ T cells in (A) (*n* = 3). (G–J) Splenic OT1^+^ CD8^+^ T cells were stimulated with various doses of N4 peptide for 6 h. Representative dot plots (G), histograms (I) and quantifications (H,J) of CD25, CD69, PS exposure, and MFI of DCF, Hoechst 33342 in splenic OT1^+^ CD8^+^ T cells (*n* = 3). Each dot represents a biological replicate (F) or one individual mouse (B,D,E,H,J). Data are presented as mean ± SEM from one representative experiment of at least two independent experiments (A to J). Statistical analysis was performed using one‐way ANOVA (H,J), or two‐sided paired Student's *t* test (D,E), or unpaired Student's *t* test (B,F). *ns*, not significant. ^*^ *p*<0.05, ^**^ *p*<0.01, ^***^ *p*<0.001.

These observations were not limited to antibody‐mediated activation. Antigen‐specific activation of OT‐I (Ovalbumin T cell) CD8^+^ T cells similarly resulted in PS exposure, increased DCF fluorescence, and enhanced Hoechst 33342 incorporation in response to both the high‐affinity peptide N4 and the low‐affinity peptide G4 (Figure 6G–J and Figure S9A–D). Comparable results were also obtained in previously activated CD8^+^ T cells upon anti‐CD3ε restimulation (Figure S9E,F), while the DNA content remained unchanged after restimulation assessed by Hoechst 33342 and PI staining of permeabilized T cells (Figure S9G,H). Although the increase in DCF signal likely reflects both elevated intracellular ROS and lipid scrambling, the consistently accelerated uptake of Hoechst 33342 across activation conditions supports the conclusion that activation‐associated scrambling state increases PM permeability to hydrophobic molecules.

### 2.8 Lipid Scrambling Regulates the PM Permeation of Hydrophobic Drugs and Hormone‐Like Molecules

The PM is the principal barrier governing cellular exposure to therapeutically and physiologically active lipophilic compounds, including drugs, steroid hormones, and lipid‐derived metabolites [3, 34, 40, 70]. These molecules are widely assumed to enter cells primarily through passive diffusion driven by concentration gradients [32, 33, 37]. However, the extent to which this process is modulated by membrane lipid organization in living cells remains poorly defined.

Given our findings that TMEM16F‐dependent lipid scrambling broadly alters the permeability of fluorescent hydrophobic probes, we next questioned whether this mechanism extends to structurally and functionally relevant bioactive molecules. To address this question, we assessed the uptake of the chemotherapeutic agent doxorubicin, the fluorescently labeled hormone 17‐β‐estradiol (Estradiol Glow), and vitamin A derivative all‐trans retinoic acid (ATRA) (Figure 7A). These compounds are fluorescently detectable by flow cytometry [74, 75]. Strikingly, TMEM16F‐mediated lipid scrambling differentially regulated the intracellular accumulation of these compounds. In WT thymocytes, induction of lipid scrambling significantly accelerated the cellular entry of 17‐β‐estradiol and doxorubicin, whereas ATRA incorporation was reduced under the same conditions (Figure 7B–D). These effects were strictly dependent on TMEM16F, as ionomycin treatment failed to alter uptake in TMEM16F‐deficient cells (Figure 7B–D). Similar TMEM16F‐dependent regulation of estradiol and ATRA uptake was observed in RMA cells (Figure 7E,F), indicating that this phenomenon is not restricted to a single cell type.

**FIGURE 7:**
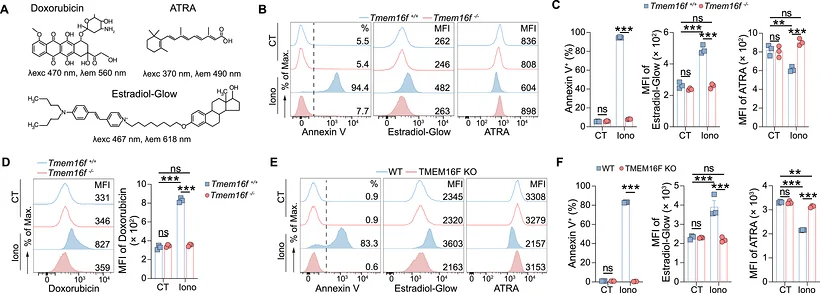
Lipid scrambling regulates the PM permeation of hydrophobic drugs and hormone‐like molecules. (A) Molecular structures of doxorubicin, all‐trans retinoic acid (ATRA) and Estradiol‐Glow. (B–D) Staining of Annexin V containing Estradiol‐Glow (1 µM), Doxorubicin (10 µM) or ATRA (20 µ) for 10 min in WT and TMEM16F KO thymocytes treated with ionomycin (5 µ) for 5 min. Cells were washed and followed by flow cytometric analysis. Representative histograms (B and left of D) and quantifications (C and right of D) of PS exposure and intracellular levels of estradiol, doxorubicin and ATRA (*n* = 3). (E,F) As in (B to D) representative histograms (E) and quantifications (F) of PS exposure and intracellular levels of estradiol or ATRA in RMA WT and TMEM16F KO cells (*n* = 3). Each dot represents a biological replicate (C,D,F). Data are represented as mean ± SEM from one representative experiment of at least two independent experiments (B to F). Statistical analysis was performed using two‐way ANOVA (C,D,F). *ns*, not significant. ^**^ *p*<0.01, ^***^ *p*<0.001.

To further establish that membrane lipid asymmetry itself is sufficient to control drug permeation, we examined cells expressing constitutively active TMEM16F mutants (D430G‐L and F518H), which induce spontaneous phospholipid scrambling in the absence of stimulation. In TMEM16F‐deficient cells, these mutants restored PS exposure and reprogrammed the uptake of both estradiol and ATRA under resting conditions (Figure S10A–F). Thus, modulation of lipid asymmetry alone is sufficient to reshape cellular exposure to pharmacologically relevant hydrophobic molecules. Although we are currently unable to predict how lipid scrambling affects the permeation of specific molecules, we have summarized the physicochemical properties of those probes used in this study in Table S1.

Collectively, these results demonstrate that PM lipid asymmetry is a key determinant of cellular permeability to bioactive lipophilic compounds. By dynamically tuning the physical state of the lipid bilayer, TMEM16F‐mediated scrambling regulates the cellular entry of drugs, hormones, and vitamin‐derived molecules, revealing a previously unrecognized layer of control over passive diffusion in living cells.

## 3 Discussion

The PM is traditionally viewed as a selective barrier whose permeability to hydrophobic molecules is largely determined by physicochemical properties such as lipophilicity and membrane composition. In this framework, passive diffusion is often considered an intrinsic property of the membrane rather than a dynamically regulated cellular process. Here, we identify plasma membrane phospholipid asymmetry as a previously unrecognized regulator of passive diffusion in living cells. By manipulating two mechanistically distinct scramblases, TMEM16F and XKR8, we demonstrate that disruption of lipid asymmetry profoundly alters the cellular entry of diverse hydrophobic molecules, including fluorescent probes, therapeutic agents, steroid hormones, and lipid‐soluble vitamins, while preserving the barrier function against membrane‐impermeable macromolecules. These findings establish membrane asymmetry as a critical determinant of molecular permeation and reveal that passive diffusion is subject to active cellular regulation.

A central implication of this work is that lipid scrambling represents a general mechanism for dynamically tuning membrane permeability. Lipid scrambling is typically induced by Ca^2^^+^ influx or caspase‐3 activation [8, 19, 22], yet both produced similar alterations in hydrophobic molecule uptake. The convergence of these independent pathways suggests that the permeability changes arise from the collapse of phospholipid asymmetry itself rather than from scramblase‐specific functions. Because lipid scrambling occurs in diverse biological contexts, including immune cell activation, apoptosis, membrane repair, cell‐cell fusion, and viral infection, regulation of membrane permeability may represent an underappreciated consequence of phospholipid redistribution across the bilayer.

Our findings further suggest that the relationship between membrane asymmetry and permeability is more complex than a simple increase or decrease in barrier function. In this study, the observed intracellular accumulation of hydrophobic molecules reflects a balance between their entry through the PM and their exit via pump‐mediated efflux and microvesicle shedding. Some hydrophobic molecules exhibited enhanced cellular accumulation following lipid scrambling, whereas others showed reduced accumulation. In contrast to Yo‐Pro‐1, which enters cells through large‐pore channels such as pannexin‐1 or through compromised PM [76, 77], these observations indicate that loss of asymmetry does not globally “open” or “close” the membrane. Instead, lipid scrambling appears to remodel the physicochemical environment of the bilayer in a molecule‐specific manner as evidenced by changes in MC540 and Laurdan signals, which preferentially bind to looser packing of the plasma membrane [56, 57, 58]. Consistent with this interpretation, scrambling was accompanied by alterations in membrane lipid packing, fluidity, and rigidity, parameters known to influence molecular partitioning and diffusion within lipid bilayers. As previously reported, scrambled membrane exhibits an approximately 80% increase in water permeability compared to asymmetrical PM [10], reinforcing the critical role of PM asymmetry in regulating membrane permeability. We therefore propose that phospholipid asymmetry contributes to the formation of a specialized membrane environment that governs both the kinetics and equilibrium of hydrophobic molecule permeation.

An unexpected observation was that certain intracellularly preloaded fluorescent probes could be lost following lipid scrambling (Figure S1K,N). Notably, experiments using preloaded CTV and Calcein‐AM, together with MDR pump inhibitors, indicated that efflux did not significantly affect probe accumulation during ionomycin treatment (Figure S1D,E). Although microvesicle shedding has been reported in ionomycin‐treated cells [27], experiments using CTV labeling and GFP‐overexpressing cells indicated that only a small fraction of cytosolic protein was lost under our experimental conditions (Figure S1F–H). Therefore, MDR pump‐mediated efflux and microvesicle shedding are unlikely to fully account for the marked reduction in intracellular fluorescence observed here. Instead, our data suggest that lipid scrambling may influence other forms of bidirectional molecular transport, affecting not only molecular entry but also intracellular retention. Reduced intracellular accumulation may therefore reflect enhanced outward diffusion in addition to altered inward permeation. This finding raises the intriguing possibility that dynamic regulation of phospholipid asymmetry contributes to the export or redistribution of endogenous hydrophobic metabolites, thereby extending the functional consequences of lipid scrambling beyond membrane signaling.

These results also have important methodological implications. Cell‐permeable fluorescent probes are foundational tools for studying intracellular processes, including redox signaling, mitochondrial function, membrane potential, and nucleic acid dynamics. The interpretation of these probes generally assumes that membrane permeability remains constant across experimental conditions. Our findings challenge this assumption. Under conditions associated with lipid scrambling, changes in fluorescence intensity may reflect altered probe permeation or retention rather than genuine changes in the biological parameter being measured. This phenomenon was particularly evident for ROS‐sensitive dyes, whose apparent reduction following ionomycin treatment contrasted with direct measurements demonstrating increased intracellular ROS production. These observations highlight the need for complementary approaches and appropriate controls when studying biological systems in which membrane asymmetry may be perturbed.

The discovery that membrane asymmetry regulates permeability to bioactive molecules has broader implications for physiology and pharmacology. Cellular uptake of steroid hormones, lipid‐soluble vitamins, and many therapeutic agents is frequently assumed to occur through passive diffusion. Our results indicate that the efficiency of this process can be substantially influenced by the asymmetry state of the plasma membrane. Consequently, cellular responsiveness to extracellular signaling molecules or pharmacological compounds may depend not only on concentration gradients and receptor availability but also on the dynamic organization of membrane lipids. Scramblase activation could therefore represent an unrecognized determinant of drug efficacy, hormone responsiveness, and metabolic adaptation.

An additional finding of this study is the apparent contribution of TMEM16F to membrane permeability under resting conditions. Although TMEM16F is classically defined as a Ca^2+^‐activated scramblase, TMEM16F‐deficient cells consistently exhibited altered permeability in the absence of overt stimulation. One possibility is that localized Ca^2+^ microdomains generate transient, low‐level scrambling events that escape detection by conventional Annexin V‐based assays. Alternatively, TMEM16F may influence membrane organization through mechanisms that are partially independent of detectable phospholipid scrambling. Resolving these possibilities will be important for understanding how membrane asymmetry is maintained and dynamically regulated under physiological conditions.

Collectively, our findings reveal a fundamental connection between PM asymmetry and molecular transport. Rather than serving solely as a structural feature of cellular membranes, phospholipid asymmetry functions as a dynamic regulator of passive diffusion. By linking scramblase activity to the permeability of biologically active hydrophobic molecules, this work provides a conceptual framework that integrates membrane organization with molecular transport. More broadly, it suggests that remodeling of lipid asymmetry may represent a general mechanism by which cells regulate their exposure to environmental signals, metabolites, and therapeutic agents. This perspective opens new avenues for understanding membrane biology and may ultimately inform strategies to manipulate drug delivery and cellular responsiveness in health and disease.

## 4 Conclusion

In this study, we uncover a previously unrecognized role for PM phospholipid asymmetry in regulating the passive diffusion of lipophilic molecules in living cells. Disruption of PM lipid asymmetry by the Ca^2+^‐activated scramblase TMEM16F or the apoptosis‐associated scramblase XKR8 profoundly alters membrane biophysical properties and modulates the permeability and intracellular accumulation of diverse hydrophobic molecules, including widely used fluorescent probes, steroid hormones, and pharmacological compounds, while preserving the barrier function of the PM. Our results suggest that passive diffusion is not merely an intrinsic property of the lipid bilayer, but can be dynamically tuned through regulated phospholipid redistribution.

## 5 Materials and Methods

### 5.1 Mice

All mice used in this study were on the C57BL/6 genetic background. Experiments were performed using age‐ and sex‐matched mice (6–12 weeks old). Each experimental group comprised 4 to 5 mice. TMEM16F KO mice and *Tmem16f‐ZsGreen* knock‐in mice have been previously reported [53]. OT‐I (C57BL/6‐Tg(TcraTcrb)1100Mjb/J) mice were obtained from The Jackson Laboratory [78]. All animals were maintained under specific pathogen‐free (SPF) conditions, and all procedures were conducted in accordance with protocols approved by the institutional Animal Care and Use Committee of Anhui Medical University (LLSC20230277).

### 5.2 Cell Culture

RMA (mouse lymphoma) and YAC‐1 (mouse thymoma) cells have been reported previously [28]. EL4 (mouse T lymphoblastoma, ATCC, TIB‐39, RRID: CVCL_0255), L1210 (mouse lymphocytic leukemia, ATCC, CCL‐219, RRID: CVCL_0382), Jurkat (human T lymphoblastoma, ATCC, TIB‐152, RRID: CVCL_0367) and HEK293T (human embryonic kidney 293, ATCC, CRL‐3216, RRID: CVCL_0063) were obtained from American Type Culture Collection (ATCC). All cells were cultured at 37°C in a humidified incubator with 5% CO_2_. RMA, YAC‐1, and Jurkat cells were cultured in RPMI 1640 medium (Gibco) supplemented with 55 µM β‐mercaptoethanol, 2 mM L‐glutamine, 10% heat‐inactivated fetal bovine serum (FBS), and 1% penicillin‐streptomycin. HEK293T, EL4, and L1210 were cultured in Dulbecco's modified Eagle's medium (DMEM, Gibco) supplemented with 10% FBS and 1% penicillin‐streptomycin. All cell lines were routinely tested and confirmed to be free of mycoplasma contamination.

### 5.3 Plasmids and Generation of Cell Lines

TMEM16F‐ and XKR8‐deficient RMA cells were generated using CRISPR‐Cas9 technology. The pSpCas9(BB)‐2A‐GFP (PX458, 48138, RRID: Addgene_48138) was used for genome editing. Gene‐specific sgRNAs targeting *Tmem16f* and *Xkr8* were designed and cloned into PX458 vector, with at least two sgRNAs per gene. The sgRNAs sequences were as follows: *Tmem16f* sgRNA1, 5’‐TGACACTCTCGTTCACCCGG‐3’; sgRNA2, 5’‐GACTCCCTCTTTTTCACCGA‐3’. *Xkr8* sgRNA1, 5’‐CCACGTCTAAGGCCACATGG‐3’; sgRNA2, 5’‐CGTACTGGACAACGGCCCAC‐3’. Plasmids were introduced into RMA cells by electroporation using the Neon NxT Electroporation System (Thermo Fisher Scientific), and GFP^+^ cells were sorted by flow cytometry (BD FACSAria III). To generate RMA DKO cell lines, PX458 plasmids containing sgRNA targeting *Xkr8* were transduced into RMA TMEM16F KO cells, followed by screening as described above. The cDNAs encoding mouse *Tmem16f* (MR211153) and *Xkr8* (MR206296) were obtained from OriGene (Rockville, MD, USA). For TMEM16F reconstitution, full‐length *Tmem16f* and its mutants (R478A, F518A, F518H, AFA, D430G‐L and FAF), fused to P2A‐mCherry or GFP, were cloned into the pFB‐Neo vector (Agilent, 217561), and introduced into RMA TMEM16F KO cells via retroviral transduction. For XKR8 reconstitution, *Xkr8* and its mutants (2DA, N371A and F381A), fused to mCherry or GFP, were inserted into the pLenti‐puro vector (39481, RRID: Addgene_39481) and delivered into RMA XKR8 KO or TMEM16F XKR8 DKO cells using lentiviral infection. GFP was introduced into RMA cells by a retroviral vector pFB‐GFP. Transduced cells were sorted based on GFP^+^ or mCherry^+^ expression (BD FACSAria III) and used for subsequent experiments.

### 5.4 Antibodies and Reagents

β‐actin monoclonal antibody (66009‐1‐Ig, RRID: AB_2687938) and GFP polyclonal antibody (50430‐2‐AP, RRID:AB_11042881) were obtained from Proteintech. Anti‐mouse TMEM16F (ab234422) was purchased from Abcam (Cambridge, UK), and anti‐human TMEM16F (HPA038958, RRID: AB_10672835) was obtained from Sigma‐Aldrich (Burlington, MA, USA). Flow cytometry antibodies against mouse CD69 (H1.2F3, 104508, RRID: AB_313111), CD25 (PC61, 101916, RRID: AB_2616762), CD45 (30‐F11, 103112, RRID: AB_312977, 103106, RRID: AB_312971), Thy‐1 (30‐H12, 105318, RRID: AB_492888), CD8 (53‐6.7, 100722, RRID: AB_312761, 100706, RRID: AB_312745), CD3ε (145‐2C11, 100340, RRID: AB_11149115), as well as the corresponding isotype controls, were purchased from BioLegend (San Diego, USA). Annexin V (640941, 640945, 640918) and DAPI (422801) were also from BioLegend.

To assess the impact of phospholipid asymmetry on the uptake of cell‐permeable dyes, various chemical compounds were used. CM‐H2DCFDA (CM‐DCF, C6827), MitoTracker Green FM (M7514), MitoTracker Red CMXRos (M7512), TMRM (T668), Dextran AF488 3000 MW (D34682), CellROX (C10422), Hoechst 33342 (H21492), Hoechst 33258 (H21491), SYTOX Green (S7020), CellTrace Violet (C34557), Calcein‐AM (C34851) and Laurdan (D250) were purchased from Invitrogen (Carlsbad, CA, USA). H2DCFDA (DCF, D6883), propidium iodide (P4170), merocyanine 540 (MC540, 323756), H_2_O_2_ (H1009) and tert‐Butyl hydroperoxide (tBHP, 458139) were purchased from Sigma‐Aldrich. DHR123 (C5313), ionomycin (B6947), Y27632 (B1293), and doxorubicin (A1832) were all from APExBIO Technology (TX, USA). Raptinal (HY‐121320), CFSE (HY‐D0938), Rhodamine 123 (HY‐D0816), Verapamil (HY‐14275), Zosuquidar (HY‐15255), and ATRA (HY‐14649) were obtained from MedChemExpress (NJ, USA). Estradiol‐Glow (PR‐958S) was obtained from Jena Bioscience (Jena, Germany). Blebbistatin (S7099) was purchased from Selleck Chemicals (TX, USA).

### 5.5 Live Cell Imaging Assay

To monitor HyPer7 localization, 1 × 10^5^ RMA cells overexpressing the indicated HyPer7 constructs were washed with PBS and seeded onto poly‐L‐ornithine‐coated (1 µg/mL) 4‐chamber glass‐bottom dishes for 30 min. Cells were then stained with anti‐Thy‐1‐AF647 antibody (BioLegend, 30‐H12, 1:1000) and PI (200 ng/mL) for 30 min. Images were acquired using a Nikon Ti2‐E confocal microscope equipped with a 60 × oil objective.

To monitor the localization of mutant XKR8, 1 × 10^5^ RMA DKO cells were seeded onto 4‐chamber dishes and stained with anti‐Thy‐1‐AF647 for 30 min. Images were acquired as described above.

To assess the microvesicle shedding and hydrophobic molecule efflux during ionomycin treatment, 1 × 10^5^ RMA cells were seeded and stained with CTV (5 µM), Calcein‐AM (1 µM), and anti‐Thy‐1‐AF647 antibody for 30 min. After washing with RPMI 1640, cells were treated with ionomycin in the presence of DCF for 10 min. GFP‐expressing cells were stained with anti‐Thy‐1‐AF647 and treated with ionomycin as described. Images were acquired as described above.

For the time‐lapse imaging assay, 1 × 10^5^ WT or TMEM16F KO RMA cells were seeded in confocal dishes and stained with anti‐Thy‐1‐AF647 (1:1000) antibody as described above. After washing with RPMI 1640, cells were treated with ionomycin in the presence of Hoechst 33342 (5 µg/mL), DCF (10 µM), and PI (200 ng/mL). Images were captured at 1‐min intervals using a Nikon confocal microscope. Fluorescence intensities in each channel were quantified using ImageJ and NIS‐Elements Viewer (Version 5.42.01).

### 5.6 T Cell Activation and Staining in Vitro

Splenic single‐cell suspensions were prepared from C57 and OT1 mice. Red blood cells were lysed, and 1 × 10^6^ splenocytes or purified CD8^+^ T cells were seeded in 96‐well U‐bottom plates and incubated in RPMI 1640 medium containing anti‐CD3ε (145‐2C11) or OVA peptides (N4 or G4) at the indicated concentrations for 6 h at 37°C. For permeability assays, cells were stained with anti‐CD8 antibody (1:1000) and Annexin V (1:500) for 15 min at room temperature (RT). To assess T cell activation, cells were stained with anti‐CD8 (1:1000), CD69 (1:1000), and CD25 (1:1000) for 20 min at RT. After staining, samples were washed with binding buffer and resuspended in 1× Annexin V binding buffer containing propidium iodide (PI), Hoechst 33342, and DCF as described above. Cells were incubated for 10 min and subsequently analyzed by flow cytometry.

### 5.7 ROS Measurements

Intracellular ROS levels were assessed using the ultrafast and ultrasensitive HyPer7 system [49], with probes targeted to distinct subcellular compartments, including the cytosol, mitochondria, and plasma membrane. For expression in RMA cells, mitochondrial, cytosolic, and plasma membrane‐targeted versions of HyPer7 (Mito‐HyPer7, HyPer7‐NES, LifeAct‐HyPer7, and HyPer7‐CAAX) were cloned into pFB‐Neo vector. The probe sequences were as follows: CAAX: 5’‐TGCATGAGCTGCAAGTGTGTGCTCTCC‐3’; NES: 5’‐CTGCCCCCCCTGGAGCGGCTGACCCTG‐3’; COX8 (Mito): 5’‐ ATGTCCGTCCTGACGCCGCTGCTGCTGCGGGGCTTGACAGGCTCGGCCCGGCGGCTCCCAGTGCCGCGCGCCAAG‐3’; Lifeact: 5’‐ ATGGGCGTGGCCGACTTGATCAAGAAGTTCGAGTCCATCTCCAAGGAGGAG‐3’. Retroviral particles were generated by co‐transfecting the HyPer7 constructs with the VSV‐G packaging plasmid into GP293 cells using polyethyleneimine (PEI). After 48 h, viral supernatants were collected, filtered through a 0.22 µm filter, and supplemented with polybrene (5 µg/mL) for transduction of RMA cells. Transduced cells were sorted based on GFP^+^ expression for subsequent experiments. To assess whether the HyPer7 variants responded to H_2_O_2_, 1 × 10^5^ RMA‐HyPer7 cells were washed with PBS and resuspended in PBS containing H_2_O_2_ (100 µM) and PI for 5 min. Flow cytometry was used to detect fluorescence emission at 530 nm upon excitation at both 488 nm and 405 nm. The ratio of fluorescence intensity (F488/F405) was calculated as the HyPer7 ratio and used as a measure of intracellular ROS levels. To measure the intracellular ROS during ionomycin treatment, 1 × 10^5^ RMA‐HyPer7 cells were washed with PBS and resuspended in 1 × Annexin V binding buffer containing ionomycin (5 µM) and PI for 5 min. Tert‐Butyl hydroperoxide (tBHP, 10 µM) was used as a positive control.

In addition, intracellular ROS levels were quantified using the fluorogenic probe CM‐H2DCFDA (CM‐DCF, 10 µM) and H2DCFDA (DCF, 10 µm). RMA cells were incubated with CM‐DCF or DCF for 30 min at 37°C, washed with PBS, and subsequently treated with ionomycin. Fluorescence intensity in the FITC channel was measured by flow cytometry and used as an indicator of intracellular ROS.

### 5.8 Measurements of Plasma Membrane Permeability Altered by Lipid Scrambling

For ionomycin treatment, RMA, EL4, YAC‐1, L1210, Jurkat cells, as well as single‐cell suspensions from the thymus or spleen, were washed twice with PBS and resuspended at 1 × 10^5^ cells per sample in Annexin V binding buffer containing ionomycin (5 µM) for 5 min. Cells were washed and stained in Annexin V binding buffer with Annexin V, PI or DAPI, and the indicated cell permeable probes (Hoechst 33342, Hoechst 33258, DCF, or others mentioned in the figures) for 5 min, followed by flow cytometric analysis on an LSR Fortessa or FACSymphony A5 (BD Biosciences). Analyses were restricted to viable cells unless otherwise indicated. The information of physicochemical properties including molecular weight, net charge, intracellular trapping, and intracellular modification was listed in Table S1.

For raptinal treatment, 1 × 10^5^ RMA cells were cultured in RPMI 1640 medium supplemented with 10% FBS, 1% penicillin‐streptomycin and 0.1% β‐ME, and treated with raptinal (10 µM) for 1 h. Cells were washed, stained with Annexin V, PI or DAPI, and the indicated probes (Hoechst 33342, Hoechst 33258 or others) for 5–10 min, and analyzed as above.

For uptake kinetics, 1 × 10^6^ RMA cells were resuspended in 500 µL Annexin V binding buffer with Annexin V, PI or DAPI and indicated probes, with or without ionomycin (5 µM). Samples were then measured by flow cytometry for 10 min on a BD FACSymphony A5 flow cytometer.

For permeabilization controls, 1 × 10^6^ RMA cells treated with ionomycin or raptinal were fixed in 2% paraformaldehyde for 15 min, permeabilized with 0.3% Triton X‐100 for 10 min at room temperature, and stained with Hoechst 33342/33258 or PI prior to flow cytometry.

For dextran leakage assay, 1 × 10^6^ WT or TMEM16F KO RMA cells were electroporated (Neon NxT, Thermo Fisher Scientific) in R buffer (N1096) with 10 µg of dextran‐A488 (D34682) in a 10 µL total volume. Cells were recovered for 24 h before analysis.

Phospholipid scrambling in mutant cell lines was assessed by PS exposure. F518H and D430G‐L cells were stained with Annexin V and PI for 20 min at RT, followed by DCF or Hoechst 33342 staining for 5 min. N371A and F381A cells were incubated in serum‐free RPMI 1640 on ice for the indicated time before Annexin V and PI staining and subsequent probe labeling.

To investigate whether the microvesicle shedding or MDR pump‐mediated efflux contributes to the alteration of membrane permeability during calcium‐mediated or apoptosis‐associated lipid scrambling, cells were preloaded with CTV (1 µM), Calcein‐AM (1 µM), or were cells overexpressing GFP protein, and subsequently treated with ionomycin, stained with DCF, and analyzed by confocal or flow cytometry as previously described. For inhibitor experiments, RMA cells were treated with verapamil (1 µM, P‐glycoprotein inhibitor) or zosuquidar (100 nM, P‐glycoprotein inhibitor) for 10 min before ionomycin treatment (5 µM, 5 min) and hydrophobic probes staining; cells were preincubated with blebbistatin (10 µM, Non‐muscle myosin II inhibitor) or Y27632 (20 µM, ROCK inhibitor) for 30 min before raptinal (10 µM, 1 h) treatment, followed by staining with DCF (10 µM) and Hoechst 33342 (5 µg/mL) for 5 min. Flow cytometry data were analyzed using FlowJo V10, and confocal images were analyzed using imageJ.

### 5.9 Flow Cytometric Efflux Assay

To investigate the efficacy of MDR pump inhibitors, 2 × 10^6^ WT cells were collected and washed twice with PBS, subsequently resuspended in RPMI 1640 containing rhodamine 123 (10 µm) and incubated for 30 min at 37°C. After washing with PBS, cells were seeded into 96‐well U‐bottom plates and incubated in RPMI 1640 medium containing verapamil (1 µM) or zosuquidar (100 nM) for the indicated time at 37°C, followed by flow cytometric analysis as described above.

### 5.10 Basal Role of TMEM16F in Membrane Permeability

To evaluate the basal contribution of TMEM16F to plasma membrane permeability, thymic single‐cell suspensions were prepared from WT (*Tmem16f* ^+/+^) and TMEM16F KO (*Tmem16f* ^−/−^) mice. WT and KO thymocytes (10^6^ per condition) were differentially labeled with anti‐CD45‐APC (1:1000) and anti‐CD45‐PE (1:1000), respectively, washed and mixed at a 1:1 ratio in the same tube. Cells were then stained with Hoechst 33342 (5 µg/mL) for 20 min at RT and analyzed by flow cytometry. To exclude potential effects of fluorophore conjugate, a dye swap strategy was performed in which WT cells were labeled with PE and KO cells with APC prior to mixing and staining as above. In parallel, 10^6^ RMA WT or TMEM16F KO cells were either labeled with anti‐CD45‐APC (1:1000) or not, respectively, and subjected to the same mixing, staining, and analysis workflow.

### 5.11 Plasma Membrane Fluidity and Rigidity Measurements

To assess plasma membrane fluidity and rigidity, 1 × 10^6^ RMA WT or TMEM16F KO cells were washed twice with PBS and stained with Laurdan dye (5 µM) and Merocyanine 540 (10 nM) for 30 min at 37°C. Cells were then exposed to low‐dose ionomycin (1 µM), followed by staining with Annexin V and DAPI. Samples were analyzed by flow cytometry.

### 5.12 Statistical Analysis

Adobe Illustrator (Version 24.2.1) was used to prepare the illustrations. Statistical analyses were performed using GraphPad Prism (Version 9.4.1). No data were excluded. Two‐sided unpaired or paired Student's *t* test, one‐way ANOVA or two‐way ANOVA with Tukey's multiple‐comparisons test was used as indicated in the figure legends. Data are presented as mean ± SEM, with n defined in the figure legends. A *p*‐value less than 0.05 was considered statistically significant.

## Author Contributions

J.P., W.W., M.Y., and N.W. conceived the project, designed the experiments, and wrote the manuscript. J.P. and W.W. conducted the experiments and data analysis. J.X. and M.Y. made the molecular cloning, viral infection, and CRISPR‐Cas9 KO cells. N.W. supervised the project.

## Conflicts of Interest

The authors declare no conflicts of interest.

## Supporting information




**Supporting File**: advs78435‐sup‐0001‐SuppMat.docx

## Data Availability

Data sharing not applicable to this article as no datasets were generated or analysed during the current study.
