# Supplementary material for: Plasma Membrane Lipid Asymmetry Dynamically Regulates Passive Diffusion
Source: Adv Sci (Weinh). 2026 Oct 25:e78435. Online ahead of print. doi: 10.1002/advs.78435 (PMC13696883; doi:10.1002/advs.78435)
Supplement: Supplementary file 1 [file ADVS-9999-e78435-s001.docx]

Supplementary Materials for

**Plasma membrane lipid asymmetry dynamically regulates passive diffusion**

Jieming Ping^1,2,#^, Wenliang Wang^2,#^, Jinnuo Xu^2^, Mengyun Yang^2,^*, and Ning Wu^1-6,^*

1. Department of Immunology, School of Basic Medicine, Tongji Medical College, Huazhong University of Science and Technology, Wuhan 430000, China;
2. The First Affiliated Hospital of Anhui Medical University and Institute of Clinical Immunology Anhui Medical University, Hefei 230032, China;
3. Innovative Institute of Tumor Immunity and Medicine (ITIM), Hefei 230032, China;
4. Cell Architecture Research Center, Tongji Medical College, Huazhong University of Science and Technology, Wuhan, China
5. Anhui Province Key Laboratory of Tumor Immune Microenvironment and Immunotherapy, Hefei 230032, China;
6. Inflammation and Immune Mediated Diseases Laboratory of Anhui Province, Anhui Medical University, Hefei 230032, China;

#These authors contributed equally

*Correspondence: [wuning118@gmail.com](mailto:wuning118@gmail.com) (N. W.), mengyun16f@163.com (M. Y.)

**This file includes:**

Figs. S1 to S11

Supplementary Table 1

**Fig. S1.** **Pre-staining of cell-permeable fluorescent probes shows little impact on lipid scrambling.** (**A**) Representative immunofluorescence images (left) showing the localization of cell-permeable dyes DCF and Hoechst 33342, and quantifications (right) of their fluorescence levels in RMA WT cells following ionomycin treatment (5 μM) for 10 min (*n* = 88 - 138). Scale bar, 10 μm. (**B**) Kinetics of Annexin V and Hoechst 33258 staining immediately after ionomycin addition for 10 min. (**C**) Splenocytes were treated with ionomycin (5 μM) and then stained with cell-permeable dyes DCF (10 μM), Hoechst 33342 (5 μg/mL) and Hoechst 33258 (5 μg/mL) as described. Representative histograms (left) and quantifications (right) of PS exposure and the intracellular levels of cell permeable dyes (*n* = 3). (**D**) RMA cells were preloaded with CellTrace Violet (1 μM) and Calcein-AM (1 μM) for 30 min, followed by ionomycin treatment and stained with DCF (10 μM) for 5 min. Representative histograms (left) and quantifications (right) of PS exposure and the intracellular levels of cell permeable dyes (*n* = 3). (**E**) As in (D), representative immunofluorescence images (left) and quantifications (right) of intracellular DCF, CTV and Calcein-AM fluorescence levels (*n* = 82 for control, *n* = 71 for ionomycin). Scale bar, 10 μm. (**F**) GFP-overexpressing RMA cells were treated with ionomycin as previously described followed by staining with CTV (1 μM) for 5 min. Representative histograms (left) and quantifications (right) of PS exposure and the intracellular levels of GFP and CTV signals (*n* = 3). (**G**) As in (F), representative immunofluorescence images (left) and quantifications (right) of intracellular GFP signal and cell volume (*n* = 71 for control, *n* = 72 for ionomycin). Scale bar, 10 μm. (**H**) Immunoblot (left) and quantification of relative intensity of GFP (right) in GFP-overexpressing cell line. β-actin served as the loading control (*n* = 3). Original blot images are provided in (fig. S11B). (**I**) RMA cells were preloaded with rhodamine 123 (10 μM) and incubated in RPMI 1640 medium containing verapamil (1 μM) or zosuquidar (100 nM) for the indicated time. Representative histogram (left) and quantification (right) of intracellular level of Rho123 (*n* = 3). (**J**) Representative histograms (left) and quantifications (right) of PS exposure and the intracellular levels of DCF and Hoechst 33342. RMA cells were pretreated with verapamil (1 μM) or zosuquidar (100 nM) for 10 min, subsequently treated with ionomycin for 5 min, followed by staining with DCF and Hoechst 33342 (*n* = 3). (**K** and **L**) RMA cells were prestained with DCF (10 μM), CellTrace Violet (1 μM), Hoechst 33342 (5 μg/mL) or Hoechst 33258 (5 μg/mL) for 30 minutes. Subsequently, cells were treated with ionomycin, stained with Annexin V and PI. Representative histograms (K) and quantifications (L) of PS exposure and the intracellular levels of cell-permeable dyes (*n* = 3). (**M**) As in (K) representative histograms (left) and quantifications (right) of PS exposure and DCF fluorescence in thymocytes (*n* = 3). (**N**) As in (K), RMA cells were preloaded with Hoechst 33342 or CTV followed by permeabilization with 0.3% Triton X-100. Representative histograms (left) and quantifications (right) of intracellular levels of Hoechst 33342 and CTV (*n* = 3). (**O**) RMA cells treated with ionomycin for 5 min, fixed and permeabilized with 2% PFA and 0.3% Triton X-100, and stained with Hoechst 33342 or Hoechst 33258. Representative histogram (left) and quantification (right) showing the intracellular level of Hoechst in permeabilized RMA cells (*n* = 3). Each dot represents a single cell (A,E,G) or one biological replicate (C,D,F,H to O). Ratiometric changes shown in (D to J) were calculated relative to the control. Data are represented as mean ± SEM pooled from three individual fields (A,E,G), or from one representative experiment out of at least two independent experiments (C to O), statistical analysis was performed using one-way ANOVA (A,E,G,I), two-way ANOVA (J) or two-sided unpaired Student’s *t* test (C,D,F,H,L, to O). *ns*, not significant. ***P*<0.01, ****P*<0.001.

**Fig. S2. Construction of HyPer7 cell lines and the responsiveness to H_2_O_2_. (A)** Schematic structure of HyPer7 plasmids encoding LifeAct-HyPer7 (actin cytoskeleton), HyPer7-CAAX (inner layer of plasma membrane), Mito-HyPer7 (COX8, mitochondria), and HyPer7-NES (cytosol). (**B**) Representative histogram shows that the percentage of HyPer7 positive cells. (**C** and **D**) RMA cells from (B) were treated with H_2_O_2_ (100 μM, 5 min). Representative dot plots (C) and quantification (D) of HyPer7 F488/F405 ratio (*n* = 3). Each dot represents one biological replicate (D). Data are presented as mean ± SEM from one representative experiment of three independent experiments (D). Statistical analysis was performed using two-sided unpaired Student’s *t* test (D). ****P*<0.001.

**Fig. S3.** **TMEM16F mediated lipid scrambling broadly alters passive diffusion of cell-permeable dyes across the PM.** (**A** to **D**) Immunoblot of TMEM16F expression in EL4 (A), YAC-1 (B), L1210 (C) and Jurkat (D) cells. β-actin served as the loading control. (**E** to **H**) Representative immunofluorescence images (E and G) and kinetics (F and H) of DCF, Hoechst 33342 fluorescence levels in RMA WT and TMEM16F KO cells treated with ionomycin (5 μM) for 20 min (*n* = 3). Scale bar, 10 μm. (**I**) Kinetics of Annexin V, DCF, Hoechst 33342 and Hoechst 33258 staining immediately after ionomycin addition for 10 min. (**J**) RMA WT and TMEM16F KO cells were pre-stained with Hoechst 33258 (5 μg/mL) for 30 min and subsequently treated with ionomycin containing Annexin V and PI. Representative histogram (left) and quantification (right) showing the intracellular level of Hoechst 33258 (*n* = 3). (**K**) Representative histograms (left) and quantifications (right) of intracellular Hoechst 33342 in permeabilized WT and TMEM16F KO cells (*n* = 3). (**L**) Immunoblot of TMEM16F expression in thymocytes. β-actin served as the loading control. (**M** and **N**) Representative histograms (M) and quantifications (N) of PS exposure and intracellular fluorescence levels of DCF, CFSE, CellROX and Hoechst 33342/33258 in WT and TMEM16F KO thymocytes (*n* = 3). Each dot represents a biological replicate (J,K and N) or one individual field of view (F and H). Original blot images are provided in (fig. S11, A,C and D). Data are represented as mean ± SEM from one representative experiment out of at least two independent experiments (E to K,M and N), statistical analysis was performed using two-way ANOVA (J,K and N). *ns*, not significant. ***P*<0.01, ****P*<0.001.

**Fig. S4. Lipid scrambling alters PM permeability in murine and human cell lines.** (**A** to **C**) WT and TMEM16F KO cells were stained with DCF following ionomycin treatment. Representative dot plots (left) and quantifications (right) of PS exposure and intracellular fluorescence levels of DCF in EL4 (A), YAC-1 (B) and L1210 (C) cells (*n* = 3). (**D** and **E**) As in (A to C), representative dot plots (left panels of D), histograms (right panels of D) and quantifications (E) showing the PS exposure, MFI of DCF, CFSE and Hoechst 33342/33258 in WT and TMEM16F KO Jurkat cells after ionomycin treatment (*n* = 3). Each dot represents one biological replicate (A to C, E). Data are represented as mean ± SEM from one representative experiment out of at least two independent experiments (A to E), statistical analysis was performed using two-way ANOVA (A to C, E). *ns*, not significant. **P*<0.05, ***P*<0.01, ****P*<0.001.

**Fig. S5. TMEM16F-mediated lipid scrambling modulates cell membrane permeability.** (**A**) Immunoblot of TMEM16F expression in RMA WT, TMEM16F KO and TMEM16F KO cells overexpressing full-length TMEM16F. β-actin served as the loading control. (**B** and **C**) Representative histograms (B) and quantifications (C) of PS exposure, DCF and Hoechst 33342 fluorescence in WT, TMEM16F KO and KO cells overexpressing full-length TMEM16F or R478A mutant following ionomycin treatment (*n* = 3). (**D**) Immunoblot of TMEM16F expression in RMA WT, TMEM16F KO and TMEM16F KO cells overexpressing R478A and F518A mutants. β-actin served as the loading control. (**E** and **F**) As in (B and C)**,** representative histograms (E) and quantifications (F) showing the PS exposure and MFI of DCF and Hoechst 33342 in WT, TMEM16F KO and KO cells overexpressing F518A mutant following ionomycin treatment (*n* = 3). (**G** and **H**) As in (B and C)**,** representative histograms (G) and quantifications (H) of PS exposure and intracellular fluorescence of CellROX (*n* = 3). (**I**) Representative histograms show the percentage of TMEM16F and its mutants fused P2A-GFP or -mCherry positive cells. Each dot represents one biological replicate (C,F and H). Original blot images are provided in (fig. S11E). Data are represented as mean ± SEM from one representative experiment out of at least two independent experiments (B and C and E to H), statistical analysis was performed using two-way ANOVA (C,F and H). *ns*, not significant. **P*<0.05, ***P*<0.01, ****P*<0.001.

**Fig. S6. Functional scramblase mediated lipid scrambling alters the membrane physical properties.** (**A**) Schematic illustration of AFA chimeras, generated by inserting the TMEM16F scrambling domain (SCRD) into the corresponding region of TMEM16A, and FAF chimeras, generated by replacing the TMEM16F SCRD with the corresponding TMEM16A region. (**B** and **C**) Representative histograms (B) and quantifications (C) of PS exposure, DCF and Hoechst 33342 fluorescence in WT, TMEM16F KO and KO cells overexpressing AFA or FAF chimeras following ionomycin treatment (*n* = 3). (**D** and **E**) Representative histograms (D) and quantification (E) of PS exposure, DCF and Hoechst 33342 fluorescence in TMEM16F KO and KO expressing F518H mutant cells (*n* = 3). (**F** and **G**) Representative histograms (F) and quantification (G) of PS exposure, DCF and Hoechst 33342 fluorescence in TMEM16F KO and KO expressing D430G-L mutant cells (*n* = 3). (**H**) Immunoblot of TMEM16F expression in RMA WT, TMEM16F KO and TMEM16F KO cells overexpressing D430G-L and F518H mutants. β-actin served as the loading control. Original blot images are provided in (fig. S11E). (**I**) Representative histograms show the percentage of TMEM16F and its mutants fused P2A-GFP or -mCherry positive cells. (**J**) Representative histogram (left) and quantification (right) of intracellular levels of Hoechst 33342 in permeabilized KO and F518H mutant cells (*n* = 3). (**K** to **N**) Gating strategy, representative histograms (K and M) and quantifications (L and N) of Laurdan dye signals and MC540 signals in RMA cells treated with ionomycin (*n* = 3). Each dot represents one biological replicate (C,E,G,J,L and N). Data are represented as mean ± SEM from one representative experiment out of at least two independent experiments (B to G and J to N), statistical analysis was performed using two-sided unpaired Student’s *t* test (E,G and J), one-way ANOVA (L and N) or two-way ANOVA (C,L and N). *ns*, not significant. **P*<0.05, ***P*<0.01, ****P*<0.001.

**Fig. S7. PM integrity is unaffected by TMEM16F mediated PLs scrambling.** (**A**) RMA WT and TMEM16F KO cells were treated with ionomycin followed by stained with TMRM for 30 min. Representative histograms (left) and quantifications (right) of PS exposure and MFI of TMRM in living cells (*n* = 3). (**B**) Representative histograms (left) and quantifications (right) of PS exposure and dextran-A488 (0.1 μg/mL) uptake in living cells following ionomycin treatment (*n* = 3). (**C**) RMA WT and TMEM16F KO cells were preloaded with dextran-A488 (1 mg/mL) via electroporation, followed by ionomycin treatment as previously described. Representative histograms (left) and quantifications (right) showing the percentage of PS exposure and dextran leakage during ionomycin treatment (*n* = 3). (**D** and **E**) A dye swap experiment was performed as previously described. RMA WT or TMEM16F KO cells were labeled with anti-CD45-APC antibody. Dye labels were swapped in the second experiment. Gating strategy (left), representative histograms (middle) and quantifications (right) of Hoechst 33342 fluorescence in WT and KO cells (*n* = 3). Each dot represents one biological replicate (A to E). Data are represented as mean ± SEM from one representative experiment out of at least two independent experiments (A to E), statistical analysis was performed using two-way ANOVA (A to C) and two-sided paired Student’s *t* test (D and E). ns, not significant. ***P*<0.01, ****P*<0.001.

**Fig. S8. Generation of XKR8-deficient cell line and the localization of XKR8.** (**A** and **B**) Representative histograms (left) and quantifications (right) of PS exposure and the intracellular levels of DCF, CTV and Hoechst 33342. RMA cells were treated with verapamil (10 μM), zosuquidar (100 nM), Blebbistatin (10 μM) or Y27632 (20 μM) for 30 min, subsequently treated with raptinal (10 μM) for 1 h, followed by staining with DCF, CTV or Hoechst 33342 for 5 min (*n* = 3). (**C** to **E**) Strategy for the generation of XKR8 KO RMA cells (C) and quantitative real-time PCR (qPCR) analysis of *Xkr8* expression in WT and XKR8 KO cells (D and E), 18S rRNA as the internal reference. (**F**) Kinetics of Hoechst 33258 fluorescence recorded for 10 min after 1 h raptinal treatment in WT and XKR8 KO cells. (**G** and **H**) RMA WT and XKR8 KO cells were stained with Annexin V, PI and Hoechst 33258 following raptinal treatment. Representative histograms, gating strategy (G) and quantifications (H) are shown (*n* = 3). (**I**) RMA WT and XKR8 KO cells were prestained with TMRM followed by raptinal treatment. Representative histograms (left) and quantifications (right) of PS exposure and MFI of TMRM in living cells (*n* = 3). (**J** and **K**) Representative immunofluorescence images of XKR8-GFP (J) and XKR8-mCherry (K) localization. Scale bar, 5 μm. (**L**) Representative histograms show the percentage of XKR8 and its mutants fused GFP or mCherry positive cells. (**M**) PS exposure in RMA WT, DKO cells and DKO cells overexpressing XKR8 N371A or F381A mutant treated with raptinal (10 μM) for 1 h. Representative histogram (left) and quantification (right) of PS exposure in living cells (*n* = 3). (**N** and **O**) Gating strategy and representative histograms (N), and quantifications (O) of PS exposure, DCF and Hoechst 33342 levels in XKR8, TMEM16F double KO (DKO) cells and DKO cells overexpressing XKR8 N371A or F381A mutants, without raptinal treatment (*n* = 3). Each dot represents one biological replicate (A,B,D,H,I,M and O). Data are represented as mean ± SEM from one representative experiment out of at least two independent experiments (A,B,D,H,I,M and O), statistical analysis was performed using two-way ANOVA (A,B,H,I and M), one-way ANOVA (H and O) or two-sided unpaired Student’s *t* test (D). *ns*, not significant. ***P*<0.01, ****P*<0.001.

**Fig. S9. Altered PM permeability during antigen-specific T cell activation and restimulation.** (**A** to **D**) Splenic OT1^+^ CD8^+^ T cells were stimulated with various doses of G4 peptide for 6 h. Representative dot plots (A), histograms (C) and quantifications (B and D) showing the percentage of CD25, CD69, PS exposure and MFI of DCF, Hoechst 33342 in splenic OT1^+^ CD8^+^ T cells (*n* = 3). (**E** and **F**) Gating strategy, representative histograms (E) and quantifications (F) of PS exposure and intracellular fluorescence of DCF and Hoechst 33342 in purified OT1^+^ CD8^+^ T cells following anti-CD3ε (1 μg/mL) restimulation for 6 h (*n* = 3). (**G** and **H**) Representative histograms (G) and quantifications (H) of intracellular Hoechst 33342 fluorescence in permeabilized purified OT1^+^ CD8^+^ T cells following anti-CD3ε (1 μg/mL) restimulation for 6 h (*n* = 3). Each dot represents one individual mouse (B and D) or a biological replicate (F and H). Data are represented as mean ± SEM from one representative experiment out of at least two independent experiments (E to H), statistical analysis was performed using one-way ANOVA (B,D and F) or two-sided unpaired Student’s *t* test (F and H). *ns*, not significant. ***P*<0.01, ****P*<0.001.

**Fig. S10. Functional scramblase mediated lipid scrambling regulates the PM permeation of hydrophobic drugs.** (**A** and **B**) Representative histograms, gating strategy (A) and quantifications (B) of PS exposure, ATRA fluorescence. Fluorescence of ATRA in Annexin V^+^ and Annexin V^-^ populations in TMEM16F KO cells overexpressing F518H or D430G-L mutants (B) (*n* = 3). (**C** to **F**) As in (A and B), representative histograms, gating strategy (C and E) and quantifications (D and F) of PS exposure and MFI of Estradiol-Glow in Annexin V^+^ and Annexin V^-^ populations in TMEM16F KO cells overexpressing F518H or D430G-L mutants (*n* = 3). Each dot represents a biological replicate (B,D and F). Data are represented as mean ± SEM from one representative experiment out of at least two independent experiments (A to F), statistical analysis was performed using one-way ANOVA (B), two-sided unpaired Student’s *t* test (D and F) or two-sided paired Student’s *t* test (B,D and F). ***P*<0.01, ****P*<0.001.

**Fig. S11. Original, uncropped, and unaltered immunoblot images.** (**A**) Original Western blots images for Fig. 3A and fig. S3L. (**B**) Original Western blots images for fig. S1H. (**C** and **D**) Original Western blots images for fig. S3, A to D. (**E**) Original Western blots images for fig. S5, A and D and fig. S6H. Molecular weight markers are indicated. l.e., long exposure; s.e., short exposure.

**Supplementary Table 1. Physicochemical properties of compounds used in this study**

| **Name** | **Molecular weight** | **Net charge** | **Permeability** | **Intracellular modification** | **Intracellular trapping** |
| --- | --- | --- | --- | --- | --- |
| CM-H2DCFDA | 577.80 g/mol | Neutral (DCFH is negatively charged) | Permeable Dye | Esterase cleavage and reaction with the intracellular thiols | Cytoplasm |
| H2DCFDA | 487.29 g/mol | Neutral (DCFH is negatively charged) | Permeable Dye | Esterase cleavage | Cytoplasm |
| MitoTracker Green FM | 671.88 g/mol | Cationic | Permeable Dye | React covalently with free thiol groups of mitochondrial proteins | Mitochondria |
| MitoTracker Red CMXRos | 531.52 g/mol | Cationic | Permeable Dye | Forming a covalent thioether bond with mitochondrial proteins | Mitochondria |
| TMRM | 500.93 g/mol | Cationic | Permeable Dye | No modification | Mitochondria |
| DHR123 | 346.38 g/mol | Neutral | Permeable Dye | Oxidation by ROS | Mitochondria |
| CellROX Deep Red | N/A | Neutral | Permeable Dye | Oxidation by ROS | Cytoplasm |
| Hoechst 33342 | 615.99 g/mol | Cationic | Permeable Dye | No modification; Binding to nuclear AT-rich DNA | Nucleus |
| Hoechst 33258 | 623.96 g/mol | Cationic | Permeable Dye | No modification; Binding to nuclear AT-rich DNA | Nucleus |
| CFSE | 557.46 g/mol | Neutral | Permeable Dye | Esterase cleavage | Cytoplasm/Nucleus/PM |
| CellTrace Violet | N/A | N/A | Permeable Dye | Cleaved by intracellular esterases and covalently bound to intracellular amines | Cytoplasm |
| Estradiol-Glow | 771.95 g/mol | Neutral | Permeable Dye | No modification; Binding to estrogen receptors | Cytoplasm/Nucleus |
| Doxorubicin | 579.98 g/mol | Cationic | Permeable Dye | No modification; Inserted into DNA | Nucleus |
| ATRA | 300.44 g/mol | Negative | Permeable Dye | Binding to nuclear RAR α-γ and PPARβ/δ | Nucleus |
| Propidium Iodide (PI) | 668.39 g/mol | Cationic | Impermeable Dye | No modification | DNA/RNA |
| Dextran AF488 3000 | 3000 g/mol | Anionic | Impermeable Dye | No modification | Cytoplasm |
